# Mutual Influence of Some Flavonoids and Classical Nonionic Surfactants on Their Adsorption and Volumetric Properties at Different Temperatures

**DOI:** 10.3390/molecules27092842

**Published:** 2022-04-29

**Authors:** Anna Taraba, Katarzyna Szymczyk, Anna Zdziennicka, Bronisław Jańczuk

**Affiliations:** Department of Interfacial Phenomena, Institute of Chemical Sciences, Faculty of Chemistry, Maria Curie-Skłodowska University in Lublin, Maria Curie-Skłodowska Sq. 3, 20-031 Lublin, Poland; anna.taraba@poczta.umcs.lublin.pl (A.T.); anna.zdziennicka@mail.umcs.pl (A.Z.); bronislaw.janczuk@poczta.umcs.lublin.pl (B.J.)

**Keywords:** flavonoids, nonionic surfactants, adsorption, micellization, surface tension, contact angle

## Abstract

Due to the increasing practical use of mixtures of flavonoids with nonionic surfactants the presented studies were based on the measurements of surface tension and conductivity of aqueous solution of the quercetin (Q) and rutin (Ru) in the mixtures with Triton X-114 (TX114) and Tween 80 (T80) as well as the contact angle of model liquids on the PTFE surface covered by the quercetin and rutin layers. Based on the obtained results components and parameters of the quercetin and rutin surface tension were determined and the mutual influence of Q and Ru in the mixtures with TX114 and T80 on their adsorption and volumetric properties were considered. It was found, among others, that based on the surface tension isotherms of the aqueous solution of the single flavonoid and nonionic surfactant, the surface tension isotherms of the aqueous solution of their mixture, the composition of the mixed monolayer at the water-air interface as well as the CMC of flavonoid + nonionic surfactant mixture can be predicted. The standard Gibbs energy, enthalpy and entropy of the adsorption and aggregation of the studied mixtures were also found, showing the mechanism of the adsorption and aggregation processes of the flavonoid + nonionic surfactant mixture.

## 1. Introduction

Flavonoids which can be found mainly in blue and red fruits as well as vegetables have many very important properties for the functioning of the human population [1,2,3,4,5,6]. Among the flavonoids, the quercetin (Q) and rutin (Ru) play the important role in their practical applications [7,8,9,10,11,12]. However, these applications are limited due, among others, to the poor solubility of the flavonoids in water. The other difficulties are associated with the lack of the flavonoids stability under the influence of temperature, pH, light and enzymes [13,14].

The literature data indicate that the flavonoids stability can be improved by the addition of the nonionic surfactants [15,16]. Of these surfactants polysorbates (Tweens) and Tritons seem to be the most proper for this purpose [17,18,19,20,21]. These nonionic surfactants are biodegradable and characterized by great physicochemical stability and large water solubility. For this reason, they are widely used in the pharmaceutical, cosmetic and even food industries. Tweens, for example are widely applied as dough conditioners, enhancers of the softness retention properties of mono- and diglycerides in the yeast-leavened bakery goods (such as bread and doughnuts) and as surfactants maintaining the emulsion stability in such products as butter, chocolate, and precooked foods [22]. These applications are connected, among others, with their adsorption and aggregation properties [23,24,25]. Flavonoids, which are poorly soluble in water, can be accumulated in surfactant micelles, particularly nonionic, as well as in the mixed interface layers. As a matter of fact, the formation of mixed layers at the interfaces results from the interface tension changes. The micellization process of nonionic surfactants in the presence of flavonoids should be also changed.

The adsorption of the surfactants and other compounds at the water-air interface and micelle formation in the bulk phase depend on the surface tension of the surfactants, additives and water. It is impossible to find the components and parameters of the Q and Ru surface tension in the literature. These components and parameters of flavonoids as well as tail and head of nonionic surfactants influence on the reduction of the water surface tension by the mixed monolayer formed at the water-air interface as well as the micelle formation in the bulk phase. The adsorption and aggregation properties of the mixture of different compounds depend on the interactions between their molecules. The intermolecular interactions of the mixture compounds can be deduced, among others, based on the surface tension isotherms. It is possible that we can describe and/or predict these isotherms.

The literature reports many systems for which the surface tension isotherms of the surfactant mixtures were mathematically described or thermodynamically predicted [26,27]. However, it is difficult to find such studies dealing with the mixtures of surfactants like Tween 80, which in the opinion of some authors has antioxidant properties [28], as well as flavonoids. Therefore, the aim of our studies was to determine the surface tension isotherms of the aqueous solution of Triton X-114 (TX114) and Tween 80 (T80) with the addition of quercetin and rutin at temperature equal to 293, 303 and 313 K. These nonionic surfactants have in their molecules, among others, the aromatic ring, oxyethylene, -CH_3_ and -OH groups. There are the big differences in the sizes of TX114 and T80 molecules and it is possible to find in the literature these sizes and contactable area, which is important for understanding the intermolecular interactions [29,30]. To explain the tendency of the flavonoid + nonionic surfactant mixtures to adsorb at the water-air interface and to form the micelles in the bulk phase the knowledge of the thermodynamic parameters is useful. For determination of these parameters the measurements of the surface tension of the aqueous solution of the flavonoid + nonionic surfactant mixtures at minimum three different temperatures is needed. For more detailed explanation of the changes of the surface tension of aqueous solution of nonionic surfactants with the addition of flavonoids as a function of surfactants concentration at the constant concentration of added flavonoids, their surface tension components and parameters were determined. Thus the contact angles of water, formamide and diiodomethane on the flavonoids layer formed on the model solid were measured. The obtained isotherms of the surface tension were considered due to their description and prediction. Based on these isotherms the critical micelle concentration (CMC) values as well as thermodynamic parameters of the adsorption and micellization were established.

## 2. Results

### 2.1. Some Physicochemical Properties of Quercetin, Rutin, TX114 and T80

The adsorption and aggregation properties of different kinds of the compounds in the aqueous media depend on the volume of their molecules, the presence of the hydrophobic and hydrophilic groups in the molecules and their arrangement as well as the contactable area of molecules dependent on their configuration and orientation. The volume of the quercetin, rutin, TX114 and T80 in the aqueous environment can be established taking into account the length of the chemical bonds, the angle between the bonds and the average distance between them and water molecules [31]. This average distance can be in the range from 1.56 to 2 Å at 293 K [32,33]. In our calculations, this distance was assumed to be 2 Å but in the case of hydrogen bonds creation as equal to 1.93 Å [31].

The earlier calculations showed that the volume of a given compound molecule can be determined based on the volume of the cube in which the molecule is inscribed or the sum of the volumes of cubes in which individual parts of more complex molecules are described [31,33]. The volume of one molecule of the studied compounds determined in such a way is the smallest for quercetin and largest for T80 (Table 1). The volumes of rutin and TX114 are comparable. To examine the reliability of the calculated volume of quercetin, rutin, TX114 and T80 molecules, their molar volume and then their density was determined (Table 1). For Ru, TX114 and T80, the density determined in a such way is close to the literature data [34,35] and in the case of Q the determined value of its density is close to that determined by us (Table 1). This fact suggests that the volumes of quercetin, rutin, TX114 and T80 molecules calculated by us are reliable. If so, it is possible to establish the contactable area of these molecules in the monolayer at the water-air interface. In fact, this area depends on the orientation of the molecules of a given compound in the monolayer.

In the case of TX114 and T80 their contactable area depends also on their molecules configuration [25,31,36,37]. Table 1 presents the values of the range of the contactable area of quercetin, rutin, TX114 and T80 molecules depending on their orientation and configuration in the monolayer at the water-air interface. The comparison of the contactable area of the molecules with that occupied by the molecules of the studied compounds in the saturated monolayer at the water-air interface can be useful for the explanation of the orientation and packing of these molecules in this monolayer. The minimal area ($A_{min}$) occupied by the molecules of quercetin, rutin, TX114 and T80 can be calculated from their maximal concentration ($\Gamma^{max}$) in the surface monolayer at the water-air interface using the expression [38]:

(1) $$A_{min}=\frac{1}{N^{max}},$$

where N is the Avogadro number.

**Figure 1:**
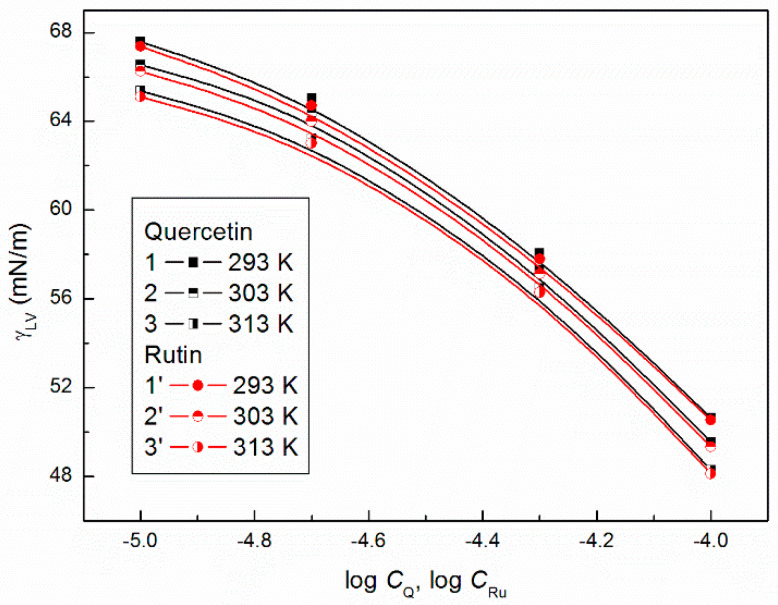
A plot of the surface tension ($\gamma_{LV}$) of the aqueous solutions of quercetin (curves 1–3) and rutin (curves 1′–3′) vs. the logarithm of their concentration (log *C*_Q_ and log *C*_Ru_) at the temperatures equal to 293 (curves 1 and 1′), 303 (curves 2 and 2′) and 313 K (curves 3 and 3′), respectively.

As it was difficult to find the $\Gamma^{max}$ values for quercetin and rutin in the literature, the surface tension ($\gamma_{LV}$) of the aqueous solution of the flavonoids was measured (Figure 1). It appeared that the obtained surface tension isotherms of the aqueous solution of quercetin and rutin can be successfully described by the numerically solved Szyszkowski equation against $\gamma_{LV}$. This equation has the form [38]:

(2) $$\gamma_{0}-\gamma_{LV}=RTn\Gamma^{max}ln\left(\frac{C}{a}+1\right),$$

where $\gamma_{0}$ is the solvent surface tension, n is the parameter used in the Gibbs isotherm equation for determination of the surface excess concentration of the given surfactant and the mixture of surfactants, C is the surfactant concentration and a is the constant.

Taking into account the values of $\Gamma^{max}$ for quercetin and rutin calculated from Equation (2) and the literature values for TX114 and T80 [23,25], the values of $A_{min}$ were determined from Equation (1) (Table 1). From the comparison of the $A_{min}$ values to those of contactable area it results that with exception for TX114 the $A_{min}$ values are close to those of the contactable area at the perpendicular orientation of their molecules towards the water-air interface (Table 1). However, in the case of TX114 it is possible that the tail of its molecules is oriented parallel towards the water-air interface. It should be mentioned that the $A_{min}$ value of TX114 is close to the contactable area of head of its molecule at the parallel orientation toward the interface (Table 1: 115.73 Å^2^ − 51.12 Å^2^ = 64.61 Å^2^).

The maximal packing of quercetin, rutin, TX114 and T80 in the saturated monolayer can be deduced based on the ratio of $\Gamma^{max}$ to $\Gamma^{\infty }$, where $\Gamma^{\infty }$ is the limiting concentration of a given compound in the saturated monolayer, which is directly associated with the size of the molecule and its orientation toward the interface. The $\Gamma^{\infty }$ values can be determined not only based on the molecule size but also using the Joos concept [39]. The ratio of $\Gamma^{max}$ to $\Gamma^{\infty }$ (Table 1) indicates that the smallest packing of TX114 in the saturated monolayer at the water-air interface, among the studied compounds, takes place. This probably results, among others, from the fact that the hydrogen ions can be joined with the oxyethylene groups in the TX114 molecules and the repulsive electrostatic interactions took place [38]. The $\Gamma^{max}$ is the reflection of the surface tension of the aqueous solution of a given surfactant. The surface tension of the solution depends on that of all its components. In the case of the compounds whose molecules have the hydrophobic and hydrophilic parts, commonly known as surfactants, their surface tension depends on the orientation of hydrophobic and hydrophilic parts toward the air phase. If the surfactant molecules are oriented by the hydrophobic part toward the air, the surface tension of surfactant is called the tail surface tension ($\gamma_{T}$) and at the orientation by the hydrophilic phase toward the air phase the head surface tension ($\gamma_{H}$) [32].

In the literature it is difficult to find the surface tension of the quercetin and rutin. Indeed, in the case of the flavonoid molecules it is impossible to distinguish the tail and head. Therefore, the components and parameters of their surface tension can be treated as an average effect of the hydrophobic and polar interactions related to the different chemical functional groups in their molecules. The Lifshitz-van der Waals component ($\gamma^{LW}$), electron-acceptor ($\gamma^{+}$) and electron-donor ($\gamma^{-}$) parameters of the studied flavonoids surface tension were determined based on the contact angle (θ) for such model liquids as water, formamide and diiodomethane on the polytetrafluoroethylene (PTFE) surface covered by the flavonoid layer (Table S1 in SM) using the van Oss et al. equation [40,41,42]:

(3) $$\gamma_{2}\left(\cos\theta +1\right)=2\left(\sqrt{\gamma_{1}^{LW}\gamma_{2}^{LW}}+\sqrt{\gamma_{1}^{+}\gamma_{2}^{-}}+\sqrt{\gamma_{1}^{-}\gamma_{2}^{+}}\right),$$

where 1 and 2 refer to the flavonoids and the nonionic surfactants, respectively.

From the calculations based on Equation (3) it appeared that the surface tension of flavonoids is almost the same as the surface tension of TX114 head and smaller than that of T80 head (Table 1). There are the big differences between the values of $\gamma^{-}$ parameter. Probably this difference has an effect on the solubility of flavonoids which is smaller than that of nonionic surfactants. The determined components and parameters of flavonoids surface tension are of significant importance in the interactions between the flavonoids and the surfactant molecules in the mixed monolayer at the water-air interface and in the micelles. It should be noted that the water-surfactant head interfacial tension has the negative values. These negative values influence on the solubility of the surfactants in water. On other hand, the positive values of the water-surfactant tail interfacial tension decide about the adsorption and aggregation properties of surfactants [38].

### 2.2. Surface Tension of the Aqueous Solution of Flavonoids with Nonionic Surfactant Mixtures

According to van Oss et al. the surface tension of solids, liquids and solutions depends on the Lifshitz-van der Waals (LW), acid-base (AB) and electrostatic (EL) intermolecular interactions in the interface region [40,41,42]. The LW interactions are present in each substance but the presence of AB and EL depend on the type of the substance. In the case of the aqueous solutions of the organic substances, whose concentration at the interface is higher than that in the bulk phase their surface tension depends on the LW, AB and/or EL intermolecular interactions between all molecules being in the interface region. As the calculated LW component of the flavonoids surface tension is higher than that of water [43], the flavonoids presence in the interface region does not reduce the water surface tension due to the LW interactions (Table 1). In the case of TX114 and T80 they can decrease of the LW component of water by the orientation of their molecules toward the air phase but to a small extent. This conclusion results from the fact that the LW component of the water surface tension is smaller than that of flavonoids and insignificantly larger than the tail surface tension of TX114 and T80 which results from only the LW interactions (Table 1).

Theoretically, the minimal surface tension of the aqueous solution of a given surfactant at its saturated monolayer at the water-air interface should be close to the surface tension of this surfactant tail. On the other hand, the largest reduction of water surface tension by the surfactant adsorption at the water-air interface takes place in the surfactant concentration range in the bulk phase corresponding to its saturated monolayer. This decrease of water surface tension results mainly from the decrease of AB component of this tension. Probably the surfactant molecules in the saturated monolayer with the increase of their concentration in the bulk phase change orientation and the part of the tail in their molecules are in the air phase as a result of the changes in the gradient of surfactants concentration in the surface region.

**Figure 2:**
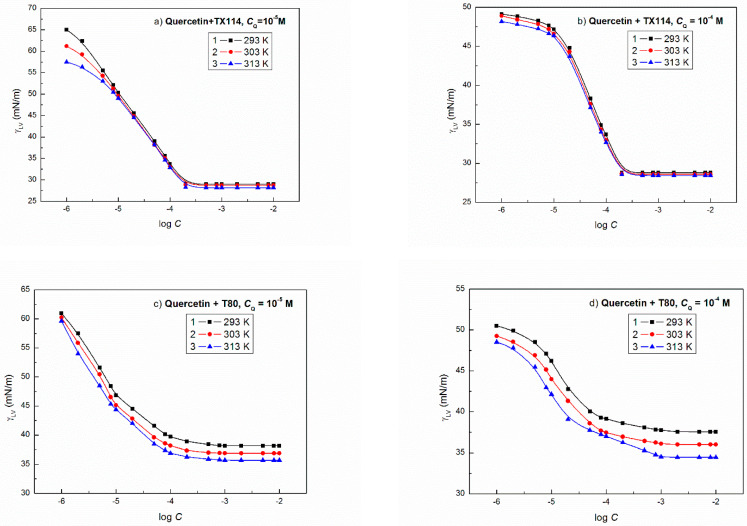
A plot of the surface tension ($\gamma_{LV}$) of the aqueous solutions of (**a**) Q + TX114 (*C*_Q_ = 10^−5^ M), (**b**) Q + TX114 (*C*_Q_ = 10^−4^ M), (**c**) Q + T80 (*C*_Q_ = 10^−5^ M) and (**d**) Q + T80 (*C*_Q_ = 10^−4^ M) vs. the logarithm of surfactant concentration (log *C*) at different temperatures equal to 293 (curve 1), 303 (curve 2), and 313 K (curve 3), respectively.

**Figure 3:**
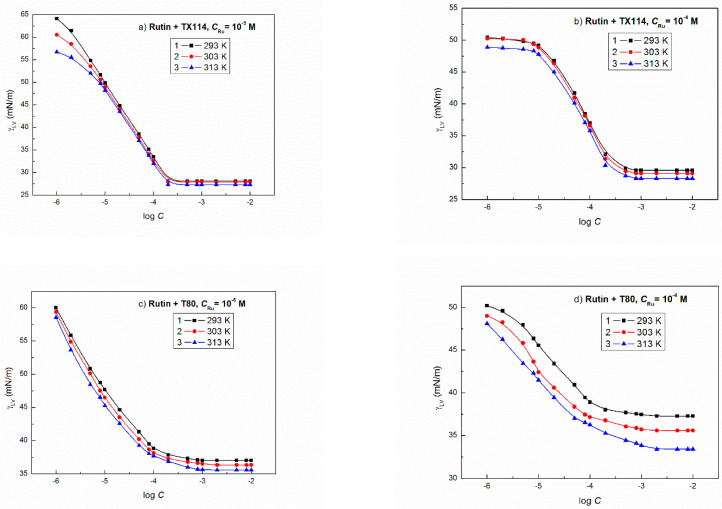
A plot of the surface tension ($\gamma_{LV}$) of the aqueous solutions of (**a**) Ru + TX114 (*C*_Ru_ = 10^−5^ M), (**b**) Ru + TX114 (*C*_Ru_ = 10^−4^ M), (**c**) Ru + T80 (*C*_Ru_ = 10^−5^ M) and (**d**) Ru + T80 (*C*_Ru_ = 10^−4^ M) vs. the logarithm of surfactant concentration (log *C*) at different temperatures equal to 293 (curve 1), 303 (curve 2), and 313 K (curve 3), respectively.

The shape of surface tension isotherm of the aqueous solution of flavonoids and nonionic surfactant mixtures proved that their concentration corresponds to the saturated mixed monolayer, particularly at the constant concentration of flavonoids equal to 1 × 10^−4^ M (Figure 2 and Figure 3). The mutual effect of the flavonoids and nonionic surfactants on the reduction of water surface tension can be clearly seen if the minimal values of $\gamma_{LV}$ of the aqueous solution of flavonoid and nonionic surfactant mixture are compared with the solution of single nonionic surfactant (Figure 2 and Figure 3) [23,25]. For almost all systems there is a linear dependence between $\gamma_{LV}$ and the temperature (Figure S1 in SM as an example) and the flavonoids cause the increase of $\gamma_{LV}$ minimal values of solution in the presence of TX114 or T80 in comparison to the solutions of single nonionic surfactants. This increase depends on the kind of flavonoid. The minimal values of $\gamma_{LV}$ for the studied flavonoid and TX114 mixture at 293 K are smaller than that of flavonoid (Table 1) and larger than the surface tension of TX114 tail (Table 1) [31]. The same relation takes place in the case of the flavonoid + T80 mixtures.

All $\;\gamma_{LV}$ isotherms obtained for the aqueous solution of flavonoids and nonionic surfactant mixtures can be successfully described by the exponential function of the second order (Figures S2–S9 in SM). This function has the form:

(4) $$\gamma_{LV}=y_{0}+A_{1}\exp\left(\frac{-C}{t_{1}}\right)+A_{2}\exp\left(\frac{-C}{t_{2}}\right),$$

where $y_{0}$, $A_{1}$, $A_{2}$, $t_{1}$ and $t_{2}$ are the constants.

The $y_{0}$ values change linearly as a function of temperature for all studied aqueous solutions of flavonoid and nonionic surfactant mixtures (Figures S10 and S11 in SM). Moreover, it seems that the $y_{0}$ values are related to the LW interactions between the water, flavonoids and nonionic surfactant molecules in the surface region. These values are close to the minimal surface tension of the solutions (Figure 2 and Figure 3). It is more difficult to find the relationship between the constants $A_{1}$, $A_{2}$, $t_{1}$ and $t_{2}$ in Equation (4) and the physicochemical properties of flavonoids and nonionic surfactants (Figures S10 and S11 in SM). Taking into account the conclusion drawn from our earlier studies [44], we can suppose that $A_{1}$ and $A_{2}$ can be joined with the polar interactions between the flavonoids and the nonionic surfactant molecules and $t_{1}$ and $t_{2}$ with the activity coefficient of the flavonoids and the nonionic surfactants in the mixed monolayer. Unfortunately, more detailed explanation of the constants in the equation of the exponential function of the second order for the studied mixtures based on the surface tension components and parameters of the water, flavonoids and nonionic surfactants is impossible.

The attempt to describe the surface tension isotherms of the aqueous solutions of a mixture of flavonoids with the nonionic surfactants by the Szyszkowski equation (Equation (2)) [38] was only partially successful (Figures S2–S9 in SM). It should be mentioned that probably only compound molecules in the monomeric form adsorbing at the water-air interface reduce the water surface tension [27]. For the studied system with T80 it was difficult to establish the surfactants concentration at which they were present in the monomeric form which was related to the flavonoids + nonionic surfactant mixtures in which the constant flavonoid concentration was equal to 1 × 10^−4^ M. The deviation of the $\gamma_{LV}$ values calculated from Equation (2) from the measured ones is greater for the aqueous solution of the mixtures of rutin with TX114 and quercetin with T80 than for the solution of the quercetin with TX114 and rutin with T80 mixtures. This is in accordance with the forces of interactions between these compounds in the bulk phase [31].

It is known that the surface tension of the aqueous solution of the binary and ternary mixtures of the surfactants can be predicted using proper methods. For this purpose the method proposed by Fainerman and Miller [45,46] is very often applied. However, this method was successfully used for prediction of the aqueous solution of surfactant mixtures surface tension in which the interactions between the mixture components were not very strong [31]. The main problem to use the Fainerman and Miller equation [45,46] for calculation of $\gamma_{LV}$ is to establish the proper area occupied by one mole of the mixture components and the mixture itself at the water-air interface ($\varpi =\frac{\pi }{RT\Gamma^{\infty }}$) particularly when the concentration of one component of the mixture is constant and the other variable. The limiting area occupied by one mole of the binary surfactant mixtures depends on the limiting surface concentration which should satisfy the following simple expression:

(5) $$\Gamma^{\infty }=\Gamma_{1}^{\infty }x_{1}^{s}+\Gamma_{2}^{\infty }x_{2}^{s},$$

where $\Gamma_{1}^{\infty }$ and $\Gamma_{2}^{\infty }$ are the limiting surface concentrations of components 1 and 2 and $x_{1}^{s}$ and $x_{2}^{s}$ are the mole fractions of surfactants 1 and 2 in the mixed monolayer at the water-air interface.

If the reduction of water surface tension by a given component of the mixed monolayer is proportional to its individual reduction at the same concentration in the aqueous solution, then $x_{1}^{s}=\frac{\pi_{1}}{\pi_{1}+\pi_{2}}$ and $x_{2}^{s}=\frac{\pi_{2}}{\pi_{1}+\pi_{2}}$ ($\pi_{1}$ and $\pi_{2}$ are the surface pressure of components 1 and 2, respectively) [44]. In such case it is possible to apply the Fainerman and Miller equation for prediction of the surface tension of the aqueous solution of binary surfactant mixtures. This equation has the form [45,46]:

(6) $$\exp\Pi =\exp\Pi_{1}+\exp\Pi_{2}-1,$$

where Π=πϖ/RT, $\Pi_{1}=\pi_{1}\varpi_{1}/RT$ and $\Pi_{2}=\pi_{2}\varpi_{2}/RT$ (*R* is the gas constant and *T* is the temperature).

The $\gamma_{LV}$ values calculated from Equation (6) for the aqueous solution of quercetin + TX114 and rutin + TX114 mixtures at the constant concentration of flavonoids equal to 1 × 10^−5^ M are very close to the measured ones (Figures S2 and S6 in SM). However, at the constant concentration of flavonoids equal to 1 × 10^−4^ M (Figures S3, S5, S7 and S9 in SM) only for the aqueous solution of quercetin with TX114 mixture the $\gamma_{LV}$ values calculated from Equation (6) are close to the measured ones but the differences between the calculated and measured $\gamma_{LV}$ values are greater than at the quercetin concentration equal to 1 × 10^−5^ M. In the case of the aqueous solution of flavonoids with T80 mixtures at the constant concentrations of flavonoids equal to 1 × 10^−5^ M (Figures S4 and S8 in SM) and to 1 × 10^−4^ M (Figures S5 and S9 in SM) there are significantly greater differences between the calculated and measured $\gamma_{LV}$ values than for the mixtures of flavonoids with TX114. The reason for these differences can be changes of the limiting concentration of flavonoids and T80. This concentration depends on the orientation of flavonoid molecules as well as the configuration of T80 ones. As mentioned above the minimal contactable area of flavonoids can be changed significantly depending on their orientation toward the water-air interface (Table 1). On the other hand, the contactable area of T80 depends largely on the configuration of its molecules in the monolayer at the water-air interface [37].

To explain the contribution of flavonoids and nonionic surfactants to reduction of water surface tension, the $\gamma_{LV}$ values for the studied systems were calculated from the following expressions:

(7) $$\gamma_{LV}=\gamma_{W}-\pi_{1}=\pi_{2},$$

and

(8) $$\gamma_{LV}=\gamma_{LV,1}x_{1}^{s}+\gamma_{LV,2}x_{2}^{s},$$

where $\gamma_{LV,1}$ and $\gamma_{LV,2}$ are the surface tension of the aqueous solution of flavonoid and nonionic surfactants, respectively at their concentration in the aqueous solution of the mixture the same as in the individual solution. Equation (7) gives reliable results only in the case when the independent adsorption takes place. It is possible at the concentration of the mixtures corresponding to the unsaturated mixed monolayer at the water-air interface.

It appeared that the shape of $\gamma_{LV}$ isotherms calculated from Equations (7) and (8) for the aqueous solution of the studied flavonoids with the TX114 mixtures is different from those for the mixtures of flavonoids with T80 (Figures S2–S9 in SM). At the constant concentration of flavonoids in the mixtures equal 1 × 10^−5^ M the independent adsorption takes place in the range of small T80 concentrations (Figures S4 and S8 in SM). For all studied systems the $\gamma_{LV}$ values calculated from Equation (8) are higher than the measured ones. This indicates that in the range of the concentrations of the flavonoids with the nonionic surfactants mixture in which independent adsorption takes place Equation (8) does not give the real results and in the concentration range of these mixtures corresponding to the saturated mixed monolayer the $\gamma_{LV,1}$ and $\gamma_{LV,2}$ values are smaller than those corresponding to mixture components surface tension values at the same concentration in their individual solutions.

### 2.3. Composition and Concentration of the Mixed Monolayer at the Water-Air Interface

The composition of the surfactants binary mixtures very often is determined using the Rosen and Hua concept [47]. Unfortunately, it is impossible to use this concept for determination of the composition of the mixed monolayers at the water-air interface containing the flavonoids and nonionic surfactants due to the difficulties to establish the range of concentration of the aqueous solution of flavonoids, nonionic surfactants and mixtures of flavonoids with nonionic surfactants at which there is the linear dependence between the surface tension of solutions and the logarithm from their concentration.

The earlier presented considerations dealing with the composition of the mixed monolayers showed that the values of the relative mole fractions of surfactants in the mixed monolayers are close to those of the fraction of the surface area occupied by a given surfactant in this monolayer [27,44]. Thus, it is possible to assume approximately that $x_{1}^{s}$ and $x_{2}^{s}$ determined in the above mentioned way can be treated as the mole fraction of flavonoid and nonionic surfactant, respectively (Figures S12 and S13 in SM). From the comparison of the $x_{2}^{s}$ with $x_{2}^{b}$ ($x_{2}^{b}=\frac{C_{2}}{C_{1}+C_{2}}$ is the molar fraction of the nonionic surfactant in the mixture in the bulk phase) it results that for all studied systems the curve of $x_{2}^{b}$ lies below that of $x_{2}^{s}$ in the concentration range of the nonionic surfactant from zero to the value at which the cross point of $x_{2}^{b}$ curve with $x_{2}^{s}$ is observed. It is possible that this point corresponds to CMC.

As a matter of fact, the composition of the mixed surface layer at the water-air interface depends on the concentration of particular components of the mixture at this interface. The concentration of the surfactants at the water-air interface is very often determined using the Gibbs isotherm equation [38]. However, for the aqueous solution of the flavonoids with the nonionic surfactant mixtures it is impossible to determine the Gibbs excess concentration for the flavonoids and in many cases for the nonionic surfactants in the whole range of their concentration in the bulk phase. The measurements of natural pH of the aqueous solution of flavonoid + nonionic surfactant mixtures indicate that its values decrease as a function of nonionic surfactants concentration (Figures S14 and S15 in SM). This suggests that the flavonoid and/or the nonionic surfactant can assume the ionic form. In the case of nonionic surfactants as mentioned above, the hydrogen ions can be joined with the oxyethylene groups and their behaviour can be similar to that of the cationic surfactants.

It should be remembered that according to the Gibbs isotherm equation [38] the excess concentration of surfactants depends on their activity in the bulk phase and on the differences of the surface tension of the solution in relation to the surfactant activity. To determine the excess Gibbs surface active ions concentration in the Gibbs equation instead of RT the nRT is used where n assumes different values depending on the kind of surfactant and mixtures of surfactants. However, the best description of the surface tension isotherms of many surfactants can be obtained from the Szyszkowski equation using the maximal Gibbs surface excess concentration determined by the use of RT in the Gibbs equation [38]. In such case the Gibbs free energy of adsorption calculated based on the constant a in the Szyszkowski equation is close to that obtained by the other methods [38]. Thus, it can be assumed that also in the Frumkin equation the same maximal values of surfactants concentration in the monolayer as in the Szyszkowski equation should be used. However, in the case of the mixture to determine the surface concentration of a given mixture component its maximal concentration which depends on the composition of the mixed monolayer as well as the contribution of this component in the reduction of water surface tension should be used. The maximum concentration of a given mixture component in the mixed monolayer at the water-air interface should be equal to the product of its molar fraction in this layer and the maximum concentration for an individual solution ($x^{s}\Gamma^{max}$). On the other hand, the contribution of a given mixture component to the reduction of the water surface tension is equal to the product of the surface pressure of the mixed monolayer (*π*) and the mole fraction of this component in the layer ($\pi_{i}=x_{i}^{s}\pi$). Taking this fact into account the Frumkin equation for i component of the surfactants mixture can assume the form:

(9) $$\pi_{i}=-RTx_{i}^{s}\Gamma_{i}^{max}ln\left(1-\frac{\Gamma_{i}}{x_{i}^{s}\Gamma_{i}^{max}}\right),$$

From the calculations made using Equation (9) it results that at the constant quercetin and rutin concentrations and variable concentration of TX114 in the bulk phase, the flavonoids concentration decreases and that of TX114 increases in the mixed monolayer at the water-air interface as a function of TX114 concentration in the bulk phase and depend on the temperature (Figures S16 and S17 in SM). In the whole range of TX114 concentration in the bulk phase at the constant flavonoids concentration equal to 1 × 10^−4^ M the saturated mixed monolayer of flavonoid and TX114 mixture is formed. The sum of flavonoid and TX114 concentrations decreases insignificantly as a function of TX114 concentration in the bulk phase, however, it is greater than for individual TX114 (Figures S16 and S17 in SM, Table 1). When the constant concentration of the flavonoid is equal to 1 × 10^−5^ M, the increase of TX114 concentration causes the increase of the sum of the flavonoid + TX114 concentrations in the mixed monolayer. For this case the sum of TX114 and flavonoid concentrations is higher than that for individual TX114. This fact proves that there are strong interactions of flavonoid molecules with TX114 ones and the area occupied by the flavonoid molecules is close to the minimal possible value (Table 1).

The behaviour of the flavonoid + T80 mixtures is somewhat different from that of flavonoid + TX114 (Figures S16 and S17 in SM). In fact, in the case of the flavonoid + T80 mixture the concentration of its components in the mixed monolayer at the water-air interface depends on the temperature and T80 variable concentration in the bulk phase. However, the sum of the flavonoid + T80 concentrations in the mixed monolayer at the constant flavonoid concentration equal to 1 × 10^−4^ M does not depend practically on the T80 concentration in the bulk phase and is higher than the maximal T80 concentration in its single monolayer [22] (Table 1, Figures S16 and S18 in SM). At the constant concentration of flavonoid equal to 1 × 10^−5^ M in the bulk phase some changes of the flavonoid + T80 concentrations sum in the mixed monolayers take place in the T80 small concentration range. For the flavonoid + TX114 and flavonoid + T80 mixtures the higher sum of concentrations in the mixed monolayer is observed at the constant flavonoid concentration in the bulk phase equal to 1 × 10^−4^ M than at 1 × 10^−5^ M. This fact confirms the conclusion that there are strong interactions between the flavonoids and nonionic surfactants in the mixed monolayer at the water-air interface and that the molecules of flavonoids occupy the minimal possible area (Table 1).

### 2.4. CMC

**Figure 4:**
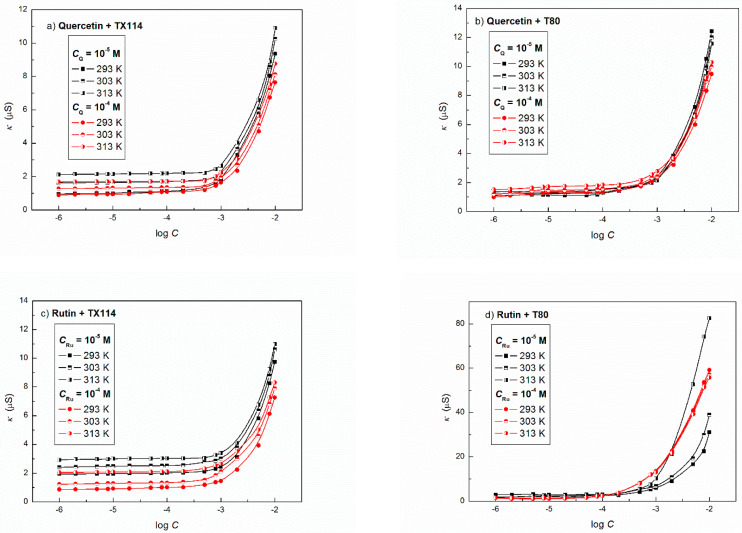
A plot of the specific conductivity (*κ*) of the aqueous solutions of (**a**) Q + TX114 (*C*_Q_ = 10^−5^ M and 10^−4^ M), (**b**) Q + T80 (*C*_Q_ = 10^−5^ M and 10^−4^ M), (**c**) Ru + TX114 (*C*_Ru_ = 10^−5^ M and 10^−4^ M) and (**d**) Ru + T80 (*C*_Ru_ = 10^−5^ M and 10^−4^ M) (**d**) vs. the logarithm of surfactant concentration (log *C*) at different temperatures equal to 293 (curve 1), 303 (curve 2), and 313 K (curve 3), respectively.

The aggregation of the nonionic surfactants in the presence of flavonoids is very important because of possible dissolution of flavonoids in the micelles. The determination of the critical micelle concentration (CMC) can be useful to understand the solubilization behaviour of flavonoids. The literature reports numerous methods for determination of CMC and among them these based on the surface tension and conductivity isotherms are often used [38]. It should be mentioned that different methods used for the CMC determination can give different values. This can be due to the fact that CMC is not related to a single concentration value of surfactants and their mixtures, but to a certain concentration range [24]. The sensitivity of a given method in determination of CMC can depend on the size and shape of the micelles or the density of the electric charge [38]. This is confirmed by the CMC values determined for the aqueous solutions of a mixture of flavonoids with the nonionic surfactants from the surface tension and conductivity isotherms (Figure 4). The CMC values determined from the conductivity isotherms of all binary mixtures of flavonoids and nonionic surfactants are higher than determined based on the surface tension isotherms (Table S2 in SM). It can be suggested that between the concentration of the aqueous solution of flavonoid + nonionic surfactant mixture at which the aggregation process was detected from the surface tension isotherm and the concentration detected from the conductivity isotherm the micelle size and/or the electric charge density may be changed. The CMC values of the aqueous solutions of the flavonoid + TX114 mixtures determined from the surface tension isotherms are insignificantly higher than those for the aqueous solution of TX114 determined also from the surface tension isotherm (Table S2 in SM) [24]. No significant effect of the constant flavonoid concentration in the bulk phase on CMC is observed. In the case of the aqueous solution of flavonoid + T80 there can be drawn the same conclusion as for the flavonoid + TX114 mixtures (Table S2 in SM). The temperature in the range from 293 to 313 K affects only insignificantly on the CMC of the studied systems. This may be due to the fact that in this temperature range the increase in the kinetic energy of the surfactant molecules can be compensated by a decrease in their hydration degree.

It is very interesting that the crossing point on the curves of the mole fraction of the TX114 and/or T80 composition in the mixed monolayer at the water-air interface with the curves of the mole fraction of these compounds in the mixture of flavonoid + nonionic surfactant in the bulk phase is close to the CMC determined from the isotherms of the surface tension (Figures S12 and S13, Table S2 in SM). This probably means that the tendency of the flavonoids to solubilize in the nonionic surfactants micelles is greater than to adsorb at the water-air interface.

### 2.5. Thermodynamic Parameters of the Adsorption and Micellization

The thermodynamic parameters of the adsorption and micellization process show the mechanism of these processes. The standard Gibbs free energy of adsorption ($\Delta G_{ads}^{0}$) and micellization ($\Delta G_{mic}^{0})$ indicate the tendency to adsorb and aggregate a given surface active agent. The standard enthalpy of adsorption ($\Delta H_{ads}^{0}$) and micellization ($\Delta H_{mic}^{0}$) provides the information about the reactions during the adsorption and micellization, respectively. Whereas the standard entropy of adsorption ($\Delta S_{ads}^{0}$) and micellization ($\Delta S_{mic}^{0}$), which is a driving force of these processes is the result of all structural changes of the bulk phase of the solution and in the interface region. The relationship between the thermodynamic function of the adsorption and micellization processes can be expressed in the forms [38,48]:

(10) $$\Delta G_{ads}^{0}=\Delta H_{ads}^{0}-T\Delta S_{ads}^{0},$$

and

(11) $$\Delta G_{mic}^{0}=\Delta H_{mic}^{0}-T\Delta S_{mic}^{0},$$

The literature reports numerous methods for determination of the thermodynamic functions [38,48]. However, in the case of the systems studied by us the most proper method for determination of $\Delta G_{ads}^{0}$ for the nonionic surfactants was based on the constant a in the Szyszkowski equation [38]. The a constant can be expressed in the form:

(12) $$a=\varpi \exp\frac{\Delta G_{ads}^{0}}{RT},$$

From the calculations of $\Delta G_{ads}^{0}$ from Equation (12) it results that the tendency of the nonionic surfactants to adsorb at the water-air interface from the bulk phase of the flavonoid + nonionic surfactant mixture is greater than in the absence of flavonoid (Table S3 in SM). This is particularly visible in the case of T80. This confirm the above mentioned suggestion that there are the strong interactions between the molecules of flavonoids and nonionic surfactants increasing its hydrophobic properties. It is not excluded that in the adsorption of nonionic surfactants at the water-air interface together with flavonoids dimmers can be formed [49].

The tendency to adsorb of flavonoid + nonionic surfactant mixture at the water-air interface can be deduced based on the $\Delta G_{ads}^{0}$ values calculated from the following equation [50]:

(13) $$\Delta G_{ads}^{0}=RT\ln\frac{CMC}{\omega }-\frac{\gamma_{W-\gamma_{LV}^{min}}}{\Gamma^{max}},$$

The values of $\Delta G_{ads}^{0}$ calculated from Equation (13) suggest that the adsorption activity of the flavonoid + nonionic surfactant mixture is higher than $\Delta G_{ads}^{0}$ of the flavonoid in the absence of the nonionic surfactant and that without flavonoid (Table S3 in SM). As follows from Table S3 for the given system there are some differences between the $\Delta G_{ads}^{0}$ values calculated from Equation (13) if the CMC values taken for calculation were determined from the surface tension isotherms, from the cross point of curves of mole fraction of nonionic surfactant in the bulk phase and in the mixed monolayers as well as those determined from the conductivity isotherms. It can be expected that the difference between the $\Delta G_{ads}^{0}$ values calculated from Equation (13) for the studied mixtures and $\Delta G_{ads}^{0}$ for the individual components of the mixture [23,25] results from the small negative values of the free energy of mixing of flavonoids and nonionic surfactants in the mixed monolayer because of strong interactions between their molecules.

Considering the influence of the interactions between the flavonoid and nonionic surfactant molecules on the tendency of the mixture of flavonoid with the surfactant to adsorb at the water-air interface, one would expect a similar tendency to form micelles whose a measure is the standard free energy of micellization. This energy can be determined from the equation which has the form [38]:

(14) $$\Delta G_{mic}^{0}=RT\ln\frac{CMC}{\omega },$$

The values of $\Delta G_{mic}^{0}$ calculated from Equation (14) for the flavonoids + nonionic surfactants mixture are practically close to that of the nonionic surfactants in the absence of flavonoids and depend on the temperature but not on the concentration of flavonoids and their type (Table S4 in SM) [24,25]. It probable means that the flavonoids do not form with nonionic surfactants typical mixed micelles but only are present in the insert of the nonionic surfactants micelles.

Due to the adsorption and micellization processes more information can be provided by the standard entropy and the standard enthalpy of adsorption and micellization. The standard entropy of adsorption and micellization can be established based on the following equations [38]:

(15) $$\frac{d\left(\Delta G_{ads}^{0}\right)}{dT}=-\Delta S_{ads}^{0},$$

and

(16) $$\frac{d\left(\Delta G_{mic}^{0}\right)}{dT}=-\Delta S_{mic}^{0}.$$

From the calculations it results that the $\Delta H_{ads}^{0}$ values for the flavonoid + TX114 mixtures are negative independently of the constant flavonoid concentration and its type. The $\Delta H_{ads}^{0}$ values for this mixture calculated from Equation (10) are close to the $\Delta H_{ads}^{0}$ values for quercetin and rutin, respectively (Table S5 in SM). This indicates that for these systems the small changes in dehydration of mixture components takes place. On contrary to the flavonoid + TX114 mixtures, the $\Delta H_{ads}^{0}$ values of the flavonoid + T80 mixtures at the constant flavonoid concentration equal to 1 × 10^−4^ M indicate the strong dehydration takes place as a result of the strong interactions between the flavonoid and the nonionic surfactant in the mixed monolayer at the water-air interface.

In the case of the micellization, in contrast to adsorption, the standard enthalpy and entropy of micellization of the flavonoids + nonionic surfactant mixtures depend on the constant flavonoids concentration and their type (Tables S6 and S7 in SM). These thermodynamic functions differ significantly from than those for TX114 and T80 in the absence of flavonoids (Table S8 in SM).

At the constant concentration of flavonoids equal to 1 × 10^−4^ M independently of the kind of nonionic surfactants, the values of $\Delta H_{mic}^{0}$ are positive. This may result from the fact that during the penetration of flavonoids molecules into the micelles significant dehydration proceeds. At the constant flavonoid concentration equal to 1 × 10^−5^ M $\Delta H_{mic}^{0}$ assumes the positive and negative values depending on the type of the systems. This fact suggests that in the micellization process the greater number of bonds is disrupted than is formed for the quercetin molecules with TX114 than with rutin. In fact, the $T\Delta S_{ads}^{0}$ and $T\Delta S_{mic}^{0}\;$(Equations (10), (11), (15) and (16)) assume larger or smaller values than $\Delta G_{ads}^{0}$ and $\Delta G_{mic}^{0}$ depending on the values of standard entropy of adsorption and micellization, respectively (Tables S3 and S4 in SM).

## 3. Materials and Methods

Quercetin (Q, ≥95%, CAS Number 117-39-5), rutin (Ru, ≥95%, CAS Number 207671-50-9), Triton X-114 (TX114, laboratory grade, CAS Number 9036-19-5) and Tween 80 (T80, CAS Number 9005-65-6) were purchased from Sigma-Aldrich and used for the solution preparation. Analytically pure ethanol (EtOH) came from POCH Gliwice. The water used for the solution preparation was doubly distilled and deionized (Destamat Bi18E). Its resistance was equal to 18.2 × 10^6^ Ω⋅m and the conductivity at T = 293 K was equal to 1.2 μS.

The Q/Ru stock solution in EtOH was prepared dissolving the Q/Ru quantity to obtain the concentration in solution equal to 2 × 10^−3^ M (*C*_Q_ or *C*_Ru_). There was also prepared the aqueous stock solution of TX114/T80, where the surfactant concentration, *C*, was equal to 1 × 10^−2^ M. Then there were made the following mixtures:

Q (C_Q_ = 1 × 10^−5^ and 1 × 10^−4^ M) + TX114 (C_TX114_ = 1 × 10^−6^–1 × 10^−2^ M)

Ru (C_Ru_ = 1 × 10^−5^ and 1 × 10^−4^ M) + TX114 (C_TX114_ = 1 × 10^−6^–1 × 10^−2^ M)

Q (C_Q_ = 1 × 10^−5^ and 1 × 10^−4^ M) + T80 (C_T80_ = 1 × 10^−6^–1 × 10^−2^ M)

Ru (C_Ru_ = 1 × 10^−5^ and 1 × 10^−4^ M) + T80 (C_T80_ = 1 × 10^−6^–1 × 10^−2^ M)

All the mixtures solution were prepared in the 100 mL glass flask wrapped with the aluminum foil, protecting flavonoids against the light. In addition, the quercetin and rutin solutions in water in the range of their concentration from 1 × 10^−5^ M to 1 × 10^−4^ M were prepared.

The stock Q and Ru solutions were also used for preparation of quercetin and rutin layers on the polytetrafluoroethylene (PTFE) surface. First, the PTFE plates were washed with a nonionic detergent and next with methanol. Next there were placed twice in an ultrasonic bath in the Milli-Q water for 15 min. Then the plates were dried with warm air for 10 min. Purity of the plates was controlled by the measurement of the water contact angle. The flavonoid layers were prepared by immersing the PTFE in the Q/Ru stock solution for 24 h. For the advancing contact angle (θ) measurements on the obtained layers water, formamide and diiodomethane were used. Water was doubly distilled and deionized (Destamat Bi18E, Inkom Instruments, Warsaw, Poland). Its resistance was equal to 18.2 × 10^6^ Ω⋅m and the conductivity at T = 293 K was equal to 1.2 μS. Formamide (>99.5%) and diiodomethane (>99%) were bought from Sigma-Aldrich.

Measurements of the advancing contact angle were made using the sessile drop method, DSA30 measuring system (Krüss, Germany) in a thermostated chamber. The contact angle was measured for at least 20 drops and good reproducibility was found. In most cases the standard deviation for each set of values was less than 1.2°.

The surface tension ($\gamma_{LV}$) measurements of the studied mixtures were made using the Krüss K100 tensiometer calibrated before the measurements. The calibration was made only at 293 K using water and methanol whose surface tension at this temperature was equal to 72.8 and 22.5 mN/m, respectively. The surface tension measurements for each concentration and composition of the mixtures were repeated at least ten times. The standard deviation of the results obtained from the measurements was ±0.1 mN/m and the uncertainty was in the range from 0.3% to 0.9%.

The conductivity and pH measurements were performed using Mettler Toledo™ Seven Multi with the accuracy ±0.5%.

All measurements were made at the temperature equal to 293, 303 and 313 K.

## 4. Conclusions

The results obtained from the measurements and their thermodynamic analysis allow to draw many conclusions.

The surface tension of quercetin and rutin are almost the same. There is an insignificant difference between their components and parameters. The contribution of the acid-base interactions to the flavonoids surface tension is insignificant, which explains their weak solubility in water. The surface tension of the flavonoids is comparable to that of the nonionic surfactants head. However, there are the great differences between the components and parameters of this tension.

The surface tension isotherms of the aqueous solution of quercetin and rutin can be described by the Szyszkowski equation. The Szyszkowski equation can be also applied for the isotherms of the surface tension of the aqueous solution of mixtures of flavonoid + nonionic surfactant in which the concentration of flavonoid is constant but that of the nonionic surfactant variable. This fact suggests that the application of the Szyszkowski equation is broader than one would expect. However, for the Szyszkowski equation application the concentration of surface active agents only in the monomeric form in aqueous solution should be taken into account.The isotherms of this solution can be also described by the exponential function of the second order. The constants in this function depend on the components and parameters of the surface active agents surface tension, however, as so far it is difficult to express them by proper mathematical relationships.

The surface tension isotherms of the aqueous solution of the flavonoid + nonionic surfactant mixture can be predicted by the Fainerman and Miller equation but not for all studied systems due to strong intermolecular interactions of the flavonoid and nonionic surfactant molecules in the mixed monolayer at the water-air interface.

The maximal concentration of the sum of flavonoid and nonionic surfactants in the mixed monolayer is higher than in the monolayer of the nonionic surfactant in the absence of flavonoid, which suggests that the attractive interactions between the flavonoids and the nonionic surfactant molecules is greater than between the nonionic surfactant molecules.

The mole fraction of flavonoid and nonionic surfactant in the mixed monolayer at the water-air interface can be determined based on the monolayer pressure of flavonoid in the absence of nonionic surfactant and the pressure of nonionic surfactant without flavonoid. The mole fraction of the nonionic surfactants in the mixed monolayer is smaller than in the bulk phase in the range of their concentrations in the bulk phase higher than CMC. The point of the intersection of the mole fraction isotherm of nonionic surfactant in the mixed monolayer with the mole fraction isotherm in the bulk phase occurs at the concentration in the bulk phase close to the CMC value determined from the surface tension isotherm of the aqueous solution of flavonoid + nonionic surfactant mixture.

The CMC of the flavonoid and nonionic surfactant mixtures determined from the surface tension isotherm is insignificantly smaller than CMC of the nonionic surfactants in the absence of flavonoids.

The CMC of the flavonoid + nonionic surfactant mixture depends only slightly on the temperature in the range from 293 to 313 K.

The standard Gibbs free energy of the adsorption and micellization of the flavonoid + nonionic surfactant mixtures indicates that the tendency to adsorb flavonoids and nonionic surfactants mixture at the water-air interface is greater than that of the surfactants in the absence of flavonoids but the tendency to form micelles is comparable.

The standard enthalpy of adsorption and micellization of the flavonoid + nonionic surfactant mixtures indicates that during the adsorption of the flavonoid + nonionic surfactant mixture the dehydration of its components in the micellization process is greater than in the adsorption except for the flavonoid + T80 mixture at the constant concentration of flavonoid equal to 1 × 10^−4^ M.

## Figures and Tables

**Table 1:** The thermodynamic parameters for quercetin, rutin, TX114 and T80.

	Quercetin	Rutin	TX114	T80
	$\Gamma^{max}$ (×10^−6^ mol/m^2^)			
T = 293 K	4.40	4.30	2.52	3.94
T = 303 K	4.30	4.15	2.45	3.81
T = 313 K	4.10	3.90	2.39	3.68
	$A_{min}$ (Å^2^)			
T = 293 K	37.73	38.61	65.89	42.14
T = 303 K	38.61	40.01	67.77	43.58
T = 313 K	40.50	42.57	69.47	45.12
	$\Gamma^{\infty }$ (×10^−6^ mol/m^2^)			
	4.77	4.70	4.65	4.04
	$A_{0}$ (Å^2^)			
	34.81	35.33	35.71	41.10
	$\Gamma^{max}{/}^{\infty }$			
	0.9224	0.9149	0.5419	0.9752
	Occupied area (Å^2^)			
	24.42–131.65	35.33–240.4	35.70–115.73 35.70–51.12	41.10–475.10 41.10–96.05
	Volume of one molecule (Å^3^)			
	456.15	832.99	856.10	1978.98
	Molar volume (cm^3^/mole)			
	274.74	501.70	515.63	1192.54
	Density (g/cm^3^)			
	1.1000 1.1100	1.2169 1.3881	1.0953 1.0580	1.0985 1.0600
	Components and parameters of surface tension (mN/m)			
$\gamma_{LV}^{LW}$	36.53	38.02	22.00 21.00	26.90 42.49
$\gamma_{LV}^{+}$	0.186	0.132	1.51	0.03
$\gamma_{LV}^{-}$	10.57	13.26	48.75	55.71
$\gamma_{LV}^{AB}$	2.80	22.65	17.16	2.59
$\gamma_{LV}$	39.33	40.67	39.16	45.08
	Water-surfactant interfacial tension (mN/m)			
Water-tail	-	-	51.00	51.00
Water-head	18.50	15.45	−13.75	−20.12

## Data Availability

Not applicable.

## Supplementary Materials

The following supporting information can be downloaded at: https://www.mdpi.com/article/10.3390/molecules27092842/s1, Table S1: The values of the contact angle (^o^) measured on the quercetin and rutin layer formed on PTFE surface for water, formamide and diiodomethane; Table S2: The values of the critical micelle concentration, CMC (M), for the flavonoid + TX114 and flavonoid + T80 mixtures at the temperature equal to 293, 303 and 313 K; Table S3:The values of the standard Gibbs free energy of adsorption ($\Delta G_{ads}^{0}$) for the flavonoid + TX114 and flavonoid + T80 mixtures at the water-air interface at the temperature equal to 293, 303 and 313 K; Table S4: The values of the standard Gibbs free energy of micellization ($\Delta G_{mic}^{0}$) for the flavonoid + TX114 and flavonoid + T80 mixtures at the water-air interface at the temperature equal to 293, 303 and 313 K. Table S5: The values of the standard Gibbs free energy of adsorption ($\Delta G_{ads}^{0}$), the standard enthalpy of adsorption ($\Delta H_{ads}^{0}$) and the standard entropy of adsorption ($\Delta S_{ads}^{0}$) for quercetin, rutin, TX114 and T80 at the temperature equal to 293, 303 and 313 K; Table S6: The values of the standard enthalpy of adsorption ($\Delta H_{ads}^{0}$) (kJ/mol) and micellization ($\Delta H_{mic}^{0}$) (kJ/mol) for the flavonoid + TX114 and flavonoid + T80 mixtures at the water-air interface at the temperature equal to 293, 303 and 313 K; Table S7: The values of the standard entropy of adsorption ($\Delta S_{ads}^{0}$) (kJ/molK) and micellization ($\Delta S_{mic}^{0}$) (kJ/molK) for the flavonoid + TX114 and flavonoid + T80 mixtures at the water-air interface at the temperature equal to 293, 303 and 313 K; Table S8: The literature values of the CMC, $\Delta G_{mic}^{0}$ (kJ/mol), $\Delta H_{mic}^{0}$ (kJ/mol) and $\Delta S_{mic}^{0}$ (kJ/molK) for TX114 and T80; Figure S1: A plot of the surface tension ($\gamma_{LV}$) of aqueous solutions of quercetin (a) and rutin (b) with TX114 and T80 (*C* = 10^−5^ M) vs. temperature (T). Curves 1 and 3 correspond to *C*_Q/Ru_ = 10^−5^ M and curves 2 and 4 correspond to *C*_Q/Ru_ = 10^−4^ M, respectively; Figure S2: A plot of the surface tension ($\gamma_{LV}$) of aqueous solutions of Q + TX114 at the constant Q concentration equal to 1 × 10^−5^ M vs. the logarithm of TX114 concentration (C) at T = 293 K (a), 303 K (b) and 313 K (c). Points 1 correspond to the measured values. Curves 2, 3, 4, 5 and 6 correspond to the values calculated from Equations (2), (4), (6), (7) and independent adsorption, respectively; Figure S3: A plot of the surface tension ($\gamma_{LV}$) of aqueous solutions of Q + TX114 at the constant Q concentration equal to 1 × 10^−4^ M vs. the logarithm of TX114 concentration (C) at T = 293 K (a), 303 K (b) and 313 K (c). Points 1 correspond to the measured values. Curves 2, 3, 4, 5 and 6 correspond to the values calculated from Equations (2), (4), (6), (7) and independent adsorption, respectively; Figure S4: A plot of the surface tension ($\gamma_{LV}$) of aqueous solutions of Q + T80 at the constant Q concentration equal to 1 × 10^−5^ M vs. the logarithm of T80 concentration (C) at T = 293 K (a), 303 K (b) and 313 K (c). Points 1 correspond to the measured values. Curves 2, 3, 4, 5 and 6 correspond to the values calculated from Equations (2), (4), (6), (7) and independent adsorption, respectively; Figure S5: A plot of the surface tension ($\gamma_{LV}$) of aqueous solutions of Q + T80 at the constant Q concentration equal to 1 × 10^−4^ M vs. the logarithm of T80 concentration (C) at T = 293 K (a), 303 K (b) and 313 K (c). Points 1 correspond to the measured values. Curves 2, 3, 4, 5 and 6 correspond to the values calculated from Equations (2), (4), (6), (7) and independent adsorption, respectively; Figure S6: A plot of the surface tension ($\gamma_{LV}$) of aqueous solutions of Ru + TX114 at the constant Ru concentration equal to 1 × 10^−5^ M vs. the logarithm of TX114 concentration (C) at T = 293 K (a), 303 K (b) and 313 K (c). Points 1 correspond to the measured values. Curves 2, 3, 4, 5 and 6 correspond to the values calculated from Equations (2), (4), (6), (7) and independent adsorption, respectively; Figure S7: A plot of the surface tension ($\gamma_{LV}$) of aqueous solutions of Ru + TX114 at the constant Ru concentration equal to 1 × 10^−4^ M vs. the logarithm of TX114 concentration (C) at T = 293 K (a), 303 K (b) and 313 K (c). Points 1 correspond to the measured values. Curves 2, 3, 4, 5 and 6 correspond to the values calculated from Equations (2), (4), (6), (7) and independent adsorption, respectively; Figure S8: A plot of the surface tension ($\gamma_{LV}$) of aqueous solutions of Ru + T80 at the constant Ru concentration equal to 1 × 10^−5^ M vs. the logarithm of T80 concentration (C) at T = 293 K (a), 303 K (b) and 313 K (c). Points 1 correspond to the measured values. Curves 2, 3, 4, 5 and 6 correspond to the values calculated from Equations (2), (4), (6), (7) and independent adsorption, respectively; Figure S9: A plot of the surface tension ($\gamma_{LV}$) of aqueous solutions of Ru + T80 at the constant Ru concentration equal to 1 × 10^−4^ M vs. the logarithm of T80 concentration (C) at T = 293 K (a), 303 K (b) and 313 K (c). Points 1 correspond to the measured values. Curves 2, 3, 4, 5 and 6 correspond to the values calculated from Equations (2), (4), (6), (7) and independent adsorption, respectively; Figure S10: A plot of the constant $y_{0}$ (a), $A_{1}\;\left(b\right),\;t_{1}\;\left(c\right),\;A_{2}\;\left(d\right)\;\mathrm{and}\;t_{2}\;\left(e\right)$ in Equation (4) for the Q + TX114 (curves 1 and 1′) and Q + T80 (curves 2 and 2′), T80 (curve 3) and TX114 (curve 4) vs. the temperature (T). Curves 1 and 2 correspond to the constant flavonoid concentration equal to 1 × 10^−5^ M, curves 1′ and 2′ to the concentration equal to 1 × 10^−4^ M; Figure S11: A plot of the constant $y_{0}$ (a), $A_{1}\;\left(b\right),\;t_{1}\;\left(c\right),\;A_{2}\;\left(d\right)\;\mathrm{and}\;t_{2}\;\left(e\right)$ in Equation (4) for the Ru + TX114 (curves 1 and 1′) and Ru +T 80 (curves 2 and 2′), T80 (curve 3) and TX114 (curve 4) vs. the temperature (T). Curves 1 and 2 correspond to the constant flavonoid concentration equal to 1 × 10^−5^ M, curves 1′ and 2′ to the concentration equal to 1 × 10^−4^ M; Figure S12: A plot of the values of $x_{2}^{b}\;$(curve 1) and $x_{2}^{s}$ at temperature equal to 293 K (curve 2), 303 K (curve 3) and 313 K (curve 4) for aqueous solutions of Q + TX114 at the constant Q concentration equal to 1 × 10^−5^ M (a) and 1 × 10^−4^ M (b) as well as Q + T80 at the constant Q concentration equal to 1 × 10^−5^ M (c) and 1 × 10^−4^ M (d) vs. the logarithm of surfactant concentration (C); Figure S13: A plot of the values of $x_{2}^{b}\;$(curve 1) and $x_{2}^{s}$ at temperature equal to 293 K (curve 2), 303 K (curve 3) and 313 K (curve 4) for aqueous solutions of Ru + TX114 at the constant Ru concentration equal to 1 × 10^−5^ M (a) and 1 × 10^−4^ M (b) as well as Ru + T80 at the constant Ru concentration equal to 1 ×10^−5^ M (c) and 1 × 10^−4^ M (d) vs. the logarithm of surfactant concentration (C); Figure S14: A plot of the values of pH of the aqueous solutions of TX114 with quercetin (curves 1 and 2) and rutin (curves 3 and 4) at temperature equal to 293 K vs. the logarithm of surfactant concentration (C); Figure S15: A plot of the values of pH of the aqueous solutions of T80 with quercetin (curves 1 and 2) and rutin (curves 3 and 4) at temperature equal to 293 K vs. the logarithm of surfactant concentration (C); Figure S16: A plot of the surface concentration (*Γ*) of Q (curves 1′ and 1″), TX114/T80 (curves 2, 2′ and 2″) and the sum values for Q and TX114/T80 (curves 3, 3′ and 3″) calculated from Equation (9) vs. the logarithm of TX114/T80 concentration (*C*) at the constant Q concentration equal to 1 × 10^−5^ M ((a) and c)) and 1 × 10^−4^ M ((b) and (d)). Curves 1, 2 and 3 correspond to temperature equal to 293 K. curves 1′, 2′ and 3′ to 303 K and curves 1″, 2″ and 3″ to 313 K, respectively; Figure S17: A plot of the surface concentration (*Γ*) of Ru (curves 1′ and 1″), TX114/T80 (curves 2, 2′ and 2″) and the sum values for Ru and TX114/T80 (curves 3, 3′ and 3″) calculated from Equation (9) vs. the logarithm of TX114/T80 concentration (*C*) at the constant Ru concentration equal to 1 × 10^−5^ M ((a) and c)) and 1 × 10^−4^ M ((b) and (d)). Curves 1, 2 and 3 correspond to temperature equal to 293 K. curves 1′, 2′ and 3′ to 303 K and curves 1″, 2″ and 3″ to 313 K, respectively.
