# Supplementary material for: Mutual Influence of Some Flavonoids and Classical Nonionic Surfactants on Their Adsorption and Volumetric Properties at Different Temperatures
Source: Molecules. 2022 Apr 29;27(9):2842. doi: 10.3390/molecules27092842 (PMC9099528; doi:10.3390/molecules27092842)
Supplement: Supplementary file 1 [file molecules-27-02842-s001.zip › molecules-1702348-supplementary.pdf]

Supplementary Materials

Mutual Influence of Some Flavonoids and Classical Nonionic Surfactants on Their Adsorption and Volumetric Properties at Different Temperatures

Anna Taraba [1], Katarzyna Szymczyk [1,*], Anna Zdziennicka [1] and Bronisław Jańczuk [1]

Department of Interfacial Phenomena, Institute of Chemical Sciences, Faculty of Chemistry, Maria Curie-Skłodowska University in Lublin, Maria Curie-Skłodowska Sq. 3, 20-031 Lublin, Poland
* Correspondence: katarzyna.szymczyk@mail.umcs.pl; Tel.: +48-81-537-55-38

Table S1. The values of the contact angle (°) measured on the quercetin and rutin layer formed on PTFE surface for water, formamide and diiodomethane.

	Water	Formamide	Diiodomethane
**Quercetin**	69.8	53.3	45.9
**Rutin**	66.1	50.2	43.1

Table S2. The values of the critical micelle concentration, CMC (M), for the flavonoid + TX114 and flavonoid + T80 mixtures at the temperature equal to 293, 303 and 313 K.

	T=293 K	T=303 K	T=313 K	T=293 K	T=303 K	T=313 K
	**Q+TX114, $C_Q = 1 \times 10^{-5}$ M**			**Q+TX114, $C_Q = 1 \times 10^{-4}$ M**		
CMC (1)	1.995×10^{-4}	1.950×10^{-4}	1.851×10^{-4}	1.905×10^{-4}	1.796×10^{-4}	1.683×10^{-4}
CMC (2)	7.968×10^{-4}	9.35×10^{-4}	9.754×10^{-4}	1.269×10^{-3}	1.116×10^{-3}	1.086×10^{-3}
CMC (3)	7.360×10^{-5}	7.755×10^{-5}	8.549×10^{-5}	2.024×10^{-4}	1.985×10^{-4}	1.970×10^{-4}
	**Q+T80, $C_Q = 1 \times 10^{-5}$ M**			**Q+T80, $C_Q = 1 \times 10^{-4}$ M**		
CMC (1)	3.890×10^{-5}	3.548×10^{-5}	4.078×10^{-5}	7.079×10^{-5}	5.623×10^{-5}	5.495×10^{-5}
CMC (2)	9.234×10^{-4}	1.056×10^{-3}	1.147×10^{-3}	1.118×10^{-3}	1.147×10^{-3}	1.179×10^{-3}
CMC (3)	6.797×10^{-5}	7.736×10^{-5}	8.568×10^{-5}	1.626×10^{-4}	1.696×10^{-4}	1.765×10^{-4}
	**Ru+TX114, $C_{Ru} = 1 \times 10^{-5}$ M**			**Ru+TX114, $C_{Ru} = 1 \times 10^{-4}$ M**		
CMC (1)	2×10^{-4}	2×10^{-4}	2×10^{-4}	2.754×10^{-4}	2.692×10^{-4}	2.428×10^{-4}
CMC (2)	1.335×10^{-3}	1.312×10^{-3}	1.288×10^{-3}	1.088×10^{-3}	8.913×10^{-4}	8.153×10^{-4}
CMC (3)	6.628×10^{-5}	7.251×10^{-5}	7.892×10^{-5}	1.989×10^{-4}	1.865×10^{-4}	1.585×10^{-4}
	**Ru+T80, $C_{Ru} = 1 \times 10^{-5}$ M**			**Ru+T80, $C_{Ru} = 1 \times 10^{-4}$ M**		
CMC (1)	1.446×10^{-4}	7.508×10^{-5}	6.918×10^{-5}	1.698×10^{-4}	1.622×10^{-4}	1.445×10^{-4}
CMC (2)	8.851×10^{-4}	9.009×10^{-4}	1.116×10^{-3}	7.125×10^{-4}	6.745×10^{-4}	6.602×10^{-4}
CMC (3)	7.616×10^{-5}	7.693×10^{-5}	7.912×10^{-5}	1.605×10^{-4}	1.783×10^{-4}	1.743×10^{-4}

Symbols 1 and 2 correspond to the CMC values determined based on the surface tension and conductivity isotherms, respectively. Symbol 3 corresponds to the CMC values obtained based on the crossing point on the curves of the mole fraction of the TX114 or T80 in the mixed monolayer at the water-air interface with the curves of the mole fraction of a given surfactant in the bulk phase.

Table S3. The values of the standard Gibbs free energy of adsorption (ΔG^0_{ads}) for the flavonoid + TX114 and flavonoid + T80 mixtures at the water-air interface at the temperature equal to 293, 303 and 313 K.

	T=293 K	T=303 K	T=313 K	T=293 K	T=303 K	T=313 K
	**Q+TX114, $C_Q = 1 \times 10^{-5}$ M**			**Q+TX114, $C_Q = 1 \times 10^{-4}$ M**		
$\Delta G^0_{ads}(1)$	-45.38	-46.77	-48.34	-45.98	-47.54	-48.56
$\Delta G^0_{ads}(2)$	-42.01	-42.82	-44.02	-41.36	-42.84	-43.71
$\Delta G^0_{ads}(3)$	-47.81	-49.09	-50.35	-45.83	-47.29	-48.15
$\Delta G^0_{ads}(4)$	-45.23	-46.77	-48.31	-38.08	-39.08	-40.43
	**Q+T80, $C_Q = 1 \times 10^{-5}$ M**			**Q+T80, $C_Q = 1 \times 10^{-4}$ M**		
$\Delta G^0_{ads}(1)$	-49.02	-50.50	-53.17	-55.02	-59.87	-63.17
$\Delta G^0_{ads}(2)$	-41.30	-41.95	-44.48	-48.17	-52.27	-55.19
$\Delta G^0_{ads}(3)$	-47.66	-48.53	-51.23	-53.00	-57.09	-60.13
$\Delta G^0_{ads}(4)$	-45.49	-47.03	-48.58	-39.33	-40.66	-43.12
	**Ru+TX114, $C_{Ru} = 1 \times 10^{-5}$ M**			**Ru+TX114, $C_{Ru} = 1 \times 10^{-4}$ M**		
$\Delta G^0_{ads}(1)$	-45.78	-47.06	-48.29	-46.3	-47.31	-49.13
$\Delta G^0_{ads}(2)$	-41.16	-42.32	-43.45	-42.95	-44.29	-45.98
$\Delta G^0_{ads}(3)$	-48.48	-49.61	-50.71	-47.09	-48.23	-50.24
$\Delta G^0_{ads}(4)$	-45.73	-47.28	-48.84	-36.26	-37.24	-38.46
	**Ru+T80, $C_{Ru} = 1 \times 10^{-5}$ M**			**Ru+T80, $C_{Ru} = 1 \times 10^{-4}$ M**		
$\Delta G^0_{ads}(1)$	-50.67	-53.16	-55.33	-59.58	-63.01	-68.19
$\Delta G^0_{ads}(2)$	-46.26	-46.90	-47.99	-56.09	-59.42	-64.24
$\Delta G^0_{ads}(3)$	-52.23	-53.10	-54.98	-59.72	-62.78	-67.70
$\Delta G^0_{ads}(4)$	-46.36	-47.94	-49.18	-40.96	-42.65	-44.32

Symbols 1, 2 and 3 corresponds to the ΔG^0_{ads} values determined from Eq. (13) taking in account the CMC (1), CMC (2) and CMC (3) values, respectively (Table S2). $\Delta G^0_{ads}(4)$ – the values calculated from Eq. (12).

Table S4. The values of the standard Gibbs free energy of micellization (ΔG^0_{mic}) for the flavonoid + TX114 and flavonoid + T80 mixtures at the water-air interface at the temperature equal to 293, 303 and 313 K.

	T=293 K	T=303 K	T=313 K	T=293 K	T=303 K	T=313 K
	**Q+TX114, $C_Q = 1 \times 10^{-5}$ M**			**Q+TX114, $C_Q = 1 \times 10^{-4}$ M**		
$\Delta G^0_{mic}(1)$	-30.54	-31.63	-32.80	-30.65	-31.84	-33.05
$\Delta G^0_{mic}(2)$	-27.16	-27.68	-28.47	-26.06	-27.23	-28.19
$\Delta G^0_{mic}(3)$	-32.96	-33.95	-34.81	-30.50	-31.58	-32.64
	**Q+T80, $C_Q = 1 \times 10^{-5}$ M**			**Q+T80, $C_Q = 1 \times 10^{-4}$ M**		
$\Delta G^0_{mic}(1)$	-34.52	-35.92	-36.73	-33.06	-34.76	-35.96
$\Delta G^0_{mic}(2)$	-26.80	-27.37	-28.05	-26.34	-27.16	-27.98
$\Delta G^0_{mic}(3)$	-33.16	-33.96	-34.80	-31.03	-31.98	-32.92
	**Ru+TX114, $C_{Ru} = 1 \times 10^{-5}$ M**			**Ru+TX114, $C_{Ru} = 1 \times 10^{-4}$ M**		
$\Delta G^0_{mic}(1)$	-30.53	-31.56	-32.60	-29.75	-30.82	-32.09
$\Delta G^0_{mic}(2)$	-26.150	-26.83	-27.75	-26.40	-27.80	-28.94
$\Delta G^0_{mic}(3)$	-33.22	-34.12	-35.02	-30.54	-31.74	-33.20
	**Ru+T80, $C_{Ru} = 1 \times 10^{-5}$ M**			**Ru+T80, $C_{Ru} = 1 \times 10^{-4}$ M**		
$\Delta G^0_{mic}(1)$	-31.32	-34.03	-35.36	-30.93	-32.09	-33.44
$\Delta G^0_{mic}(2)$	-26.91	-27.77	-28.12	-27.34	-28.50	-29.49
$\Delta G^0_{mic}(3)$	-32.88	-33.97	-35.07	-31.08	-31.85	-32.96

Symbols 1, 2 and 3 corresponds to the ΔG^0_{mic} values determined from Eq. (14) taking in account the CMC (1), CMC (2) and CMC (3) values, respectively (Table S2).

Table S5. The values of the standard Gibbs free energy of adsorption (ΔG^0_{ads}), the standard enthalpy of adsorption (ΔH^0_{ads}) and the standard entropy of adsorption (ΔS^0_{ads}) for quercetin, rutin, TX114 and T80 at the temperature equal to 293, 303 and 313 K.

	**Quercetin**	**Rutin**	**TX114**	**T80**
		ΔG^0_{ads} (kJ/mol)		
T = 293 K	-36.68	-36.87	-43.70	-32.76
T = 303 K	-37.85	-38.07	-45.36	-34.66
T = 313 K	-39.01	-39.17	-47.00	-35.01
		ΔH^0_{ads} (kJ/mol)		
T = 293 K	-2.55	-3.17	4.65	0.24
T = 303 K	-2.55	-3.17	4.65	0.24
T = 313 K	-2.55	-3.17	4.65	0.24
		ΔS^0_{ads} (kJ/molK)		
T = 293 K	0.116	0.115	0.165	0.113
T = 303 K	0.116	0.115	0.165	0.113
T = 313 K	0.116	0.115	0.165	0.113

Table S6. The values of the standard enthalpy of adsorption (ΔH^0_{ads}) (kJ/mol) and micellization (ΔH^0_{mic}) (kJ/mol) for the flavonoid + TX114 and flavonoid + T80 mixtures at the water-air interface at the temperature equal to 293, 303 and 313 K.

	T = 293 K	T = 303 K	T = 313 K	T = 293 K	T = 303 K	T = 313 K
	**Q+TX114, $C_Q = 1 \times 10^{-5}$ M**			**Q+TX114, $C_Q = 1 \times 10^{-4}$ M**		
ΔH^0_{ads}	-0.258	-0.108	-0.138	-3.653	-3.478	-3.653
ΔH^0_{mic}	2.569	2.609	2.569	4.510	4.521	4.510
	**Q+T80, $C_Q = 1 \times 10^{-5}$ M**			**Q+T80, $C_Q = 1 \times 10^{-4}$ M**		
ΔH^0_{ads}	-0.222	-0.217	-0.222	16.194	16.759	16.194
ΔH^0_{mic}	-2.144	-2.439	-2.144	9.425	9.175	9.425
	**Ru+TX114, $C_{Ru} = 1 \times 10^{-5}$ M**			**Ru+TX114, $C_{Ru} = 1 \times 10^{-4}$ M**		
ΔH^0_{ads}	-0.169	-0.164	-0.169	-4.030	-3.910	-4.030
ΔH^0_{mic}	-0.205	-0.200	-0.205	4.531	4.631	4.531
	**Ru+T80, $C_{Ru} = 1 \times 10^{-5}$ M**			**Ru+T80, $C_{Ru} = 1 \times 10^{-4}$ M**		
ΔH^0_{ads}	-5.047	-5.217	-5.047	8.264	8.254	8.264
ΔH^0_{mic}	7.649	6.269	6.269	5.842	5.937	5.842

Table S7. The values of the standard entropy of adsorption (ΔS^0_{ads}) (kJ/molK) and micellization (ΔS^0_{mic}) (kJ/molK) for the flavonoid+TX114 and flavonoid+T80 mixtures at the water-air interface at the temperature equal to 293, 303 and 313 K.

	**Q+TX114**<br>$C_Q = 1 \times 10^{-5}$ M	**Q+TX114**<br>$C_Q = 1 \times 10^{-4}$ M	**Q+T80**<br>$C_Q = 1 \times 10^{-5}$ M	**Q+T80**<br>$C_Q = 1 \times 10^{-4}$ M
ΔS^0_{ads}	0.154	0.118	0.155	0.190
ΔS^0_{mic}	0.013	0.12	0.111	0.145
	**Ru+TX114**<br>$C_{Ru} = 1 \times 10^{-5}$ M	**Ru+TX114**<br>$C_{Ru} = 1 \times 10^{-4}$ M	**Ru+T80**<br>$C_{Ru} = 1 \times 10^{-5}$ M	**Ru+T80**<br>$C_{Ru} = 1 \times 10^{-4}$ M
ΔS^0_{ads}	0.156	0.110	0.141	0.168
ΔS^0_{mic}	0.104	0.117	0.133	0.126

Table S8. The literature values of the CMC, ΔG^0_{mic} (kJ/mol), ΔH^0_{mic} (kJ/mol) and ΔS (kJ/molK) for TX114 and T80.

	CMC	ΔG^0_{mic}	ΔH^0_{mic}	ΔS^0_{mic}	Ref.
			**TX114**		
T = 293 K	1.68×10^{-4}	-30.95			
T = 303 K	1.53×10^{-4}	-32.24	6.43	0.128	[24]
T = 313 K	1.41×10^{-4}	-33.51			
			**T80**		
T = 293 K	5.74×10^{-4}	-27.96	0.75		
T = 303 K	4.41×10^{-4}	-29.57	0.12	0.098	[25]
T = 313 K	4.39×10^{-4}	-30.55	0.20		

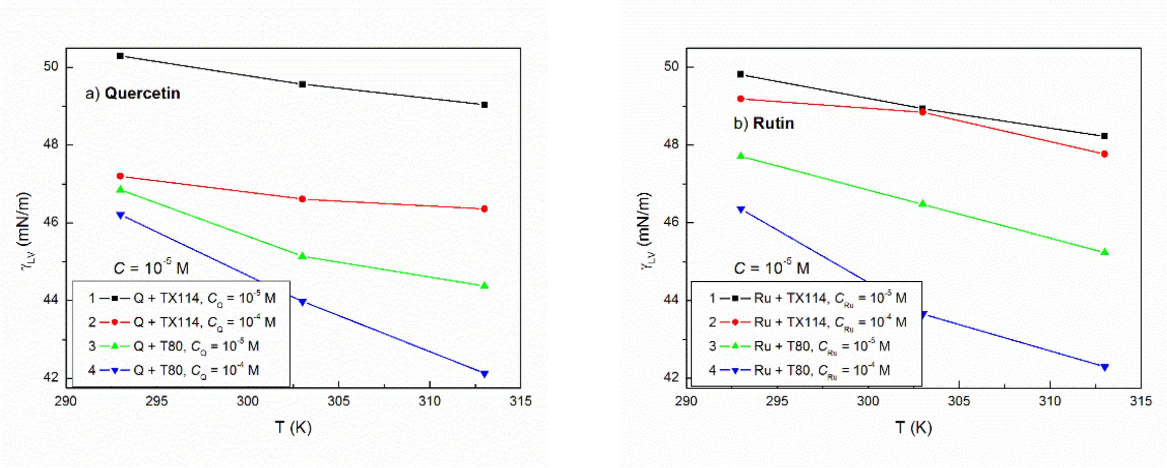

Figure S1. A plot of the surface tension (γ_{LV}) of aqueous solutions of quercetin (a) and rutin (b) with TX114 and T80 ($C = 10^{-5}$ M) vs. temperature (T). Curves 1 and 3 correspond to $C_{Q/Ru} = 10^{-5}$ M and curves 2 and 4 correspond to $C_{Q/Ru} = 10^{-4}$ M, respectively.

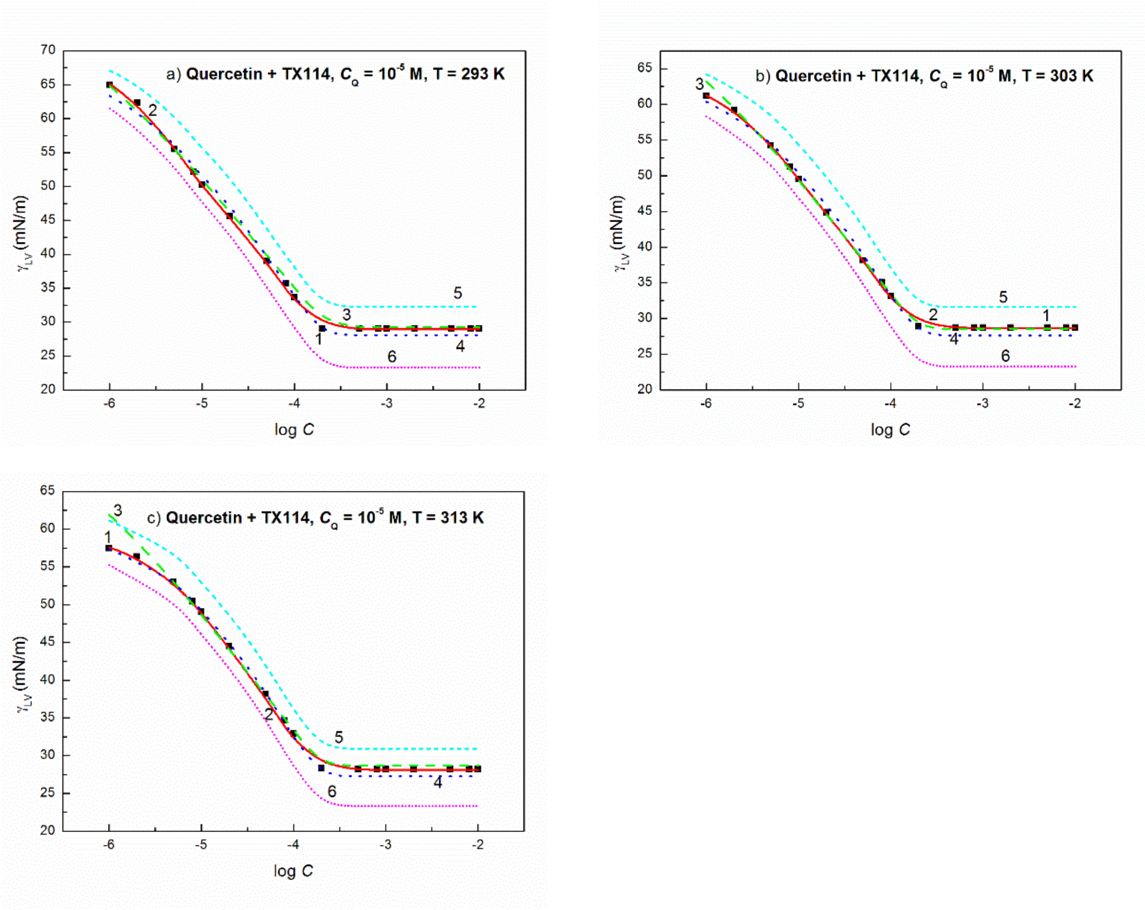

Figure S2. A plot of the surface tension (γ_{LV}) of aqueous solutions of Q + TX114 at the constant Q concentration equal to 1×10^{-5} M vs. the logarithm of TX114 concentration (C) at T = 293 K (a), 303 K (b) and 313 K (c). Points 1 correspond to the measured values. Curves 2, 3, 4, 5 and 6 correspond to the values calculated from Eqs. (4), (2), (6), (7) and independent adsorption, respectively.

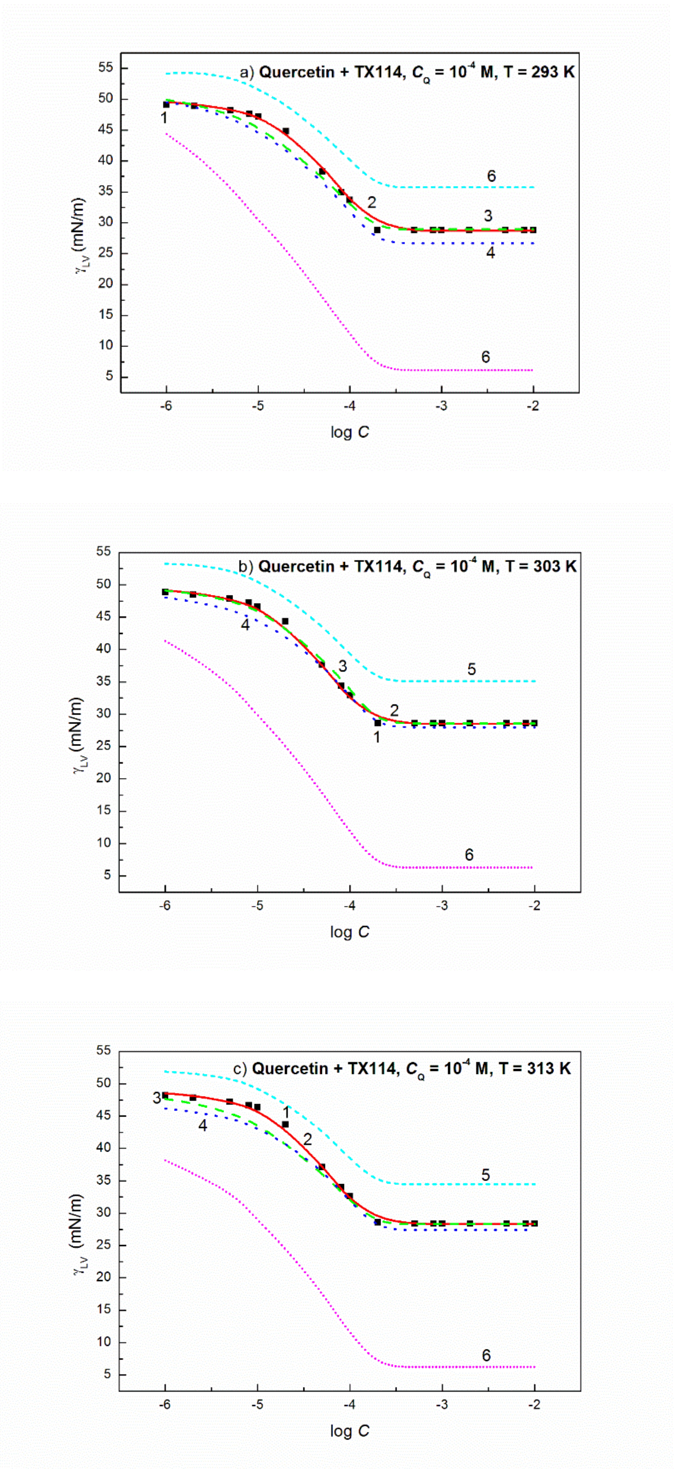

Figure S3. A plot of the surface tension (γ_{LV}) of aqueous solutions of Q + TX114 at the constant Q concentration equal to 1 × 10^{-4} M vs. the logarithm of TX114 concentration (C) at T = 293 K (a), 303 K (b) and 313 K (c). Points 1 correspond to the measured values. Curves 2, 3, 4, 5 and 6 correspond to the values calculated from Eqs. (4), (2), (6), (7) and independent adsorption, respectively.

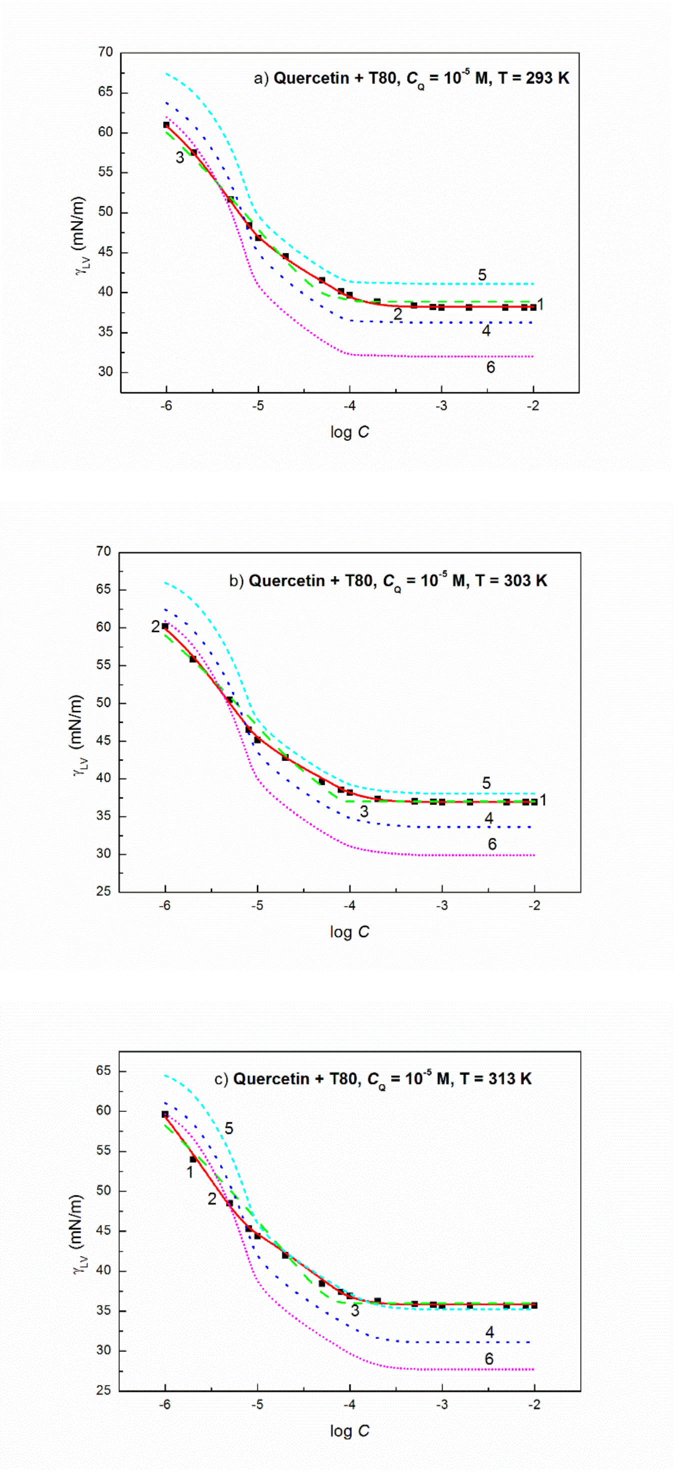

Figure S4. A plot of the surface tension (γ_{LV}) of aqueous solutions of Q + T80 at the constant Q concentration equal to 1 × 10^{-5} M vs. the logarithm of T80 concentration (C) at T = 293 K (a), 303 K (b) and 313 K (c). Points 1 correspond to the measured values. Curves 2, 3, 4, 5 and 6 correspond to the values calculated from Eqs. (4), (2), (6), (7) and independent adsorption, respectively.

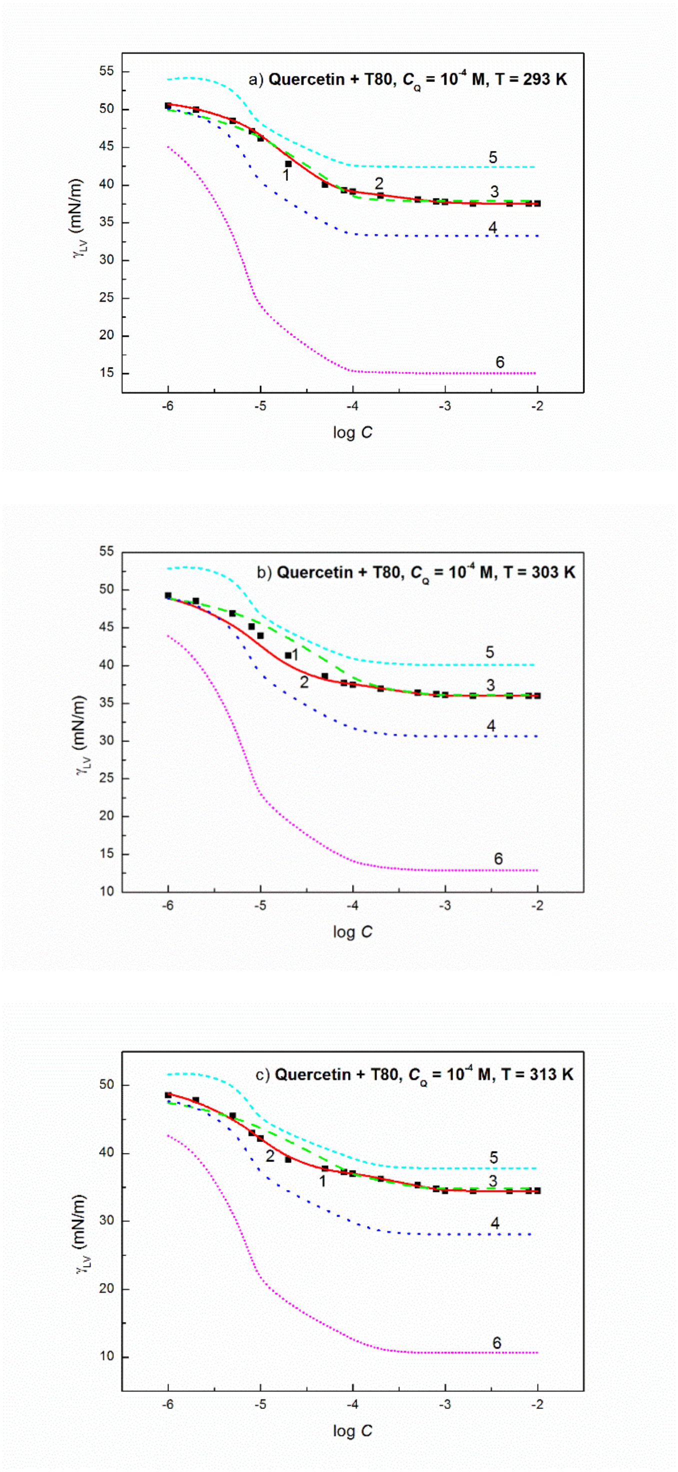

Figure S5. A plot of the surface tension (γ_{LV}) of aqueous solutions of Q + T80 at the constant Q concentration equal to 1 × 10^{-4} M vs. the logarithm of T80 concentration (C) at T = 293 K (a), 303 K (b) and 313 K (c). Points 1 correspond to the measured values. Curves 2, 3, 4, 5 and 6 correspond to the values calculated from Eqs. (4), (2), (6), (7) and independent adsorption, respectively.

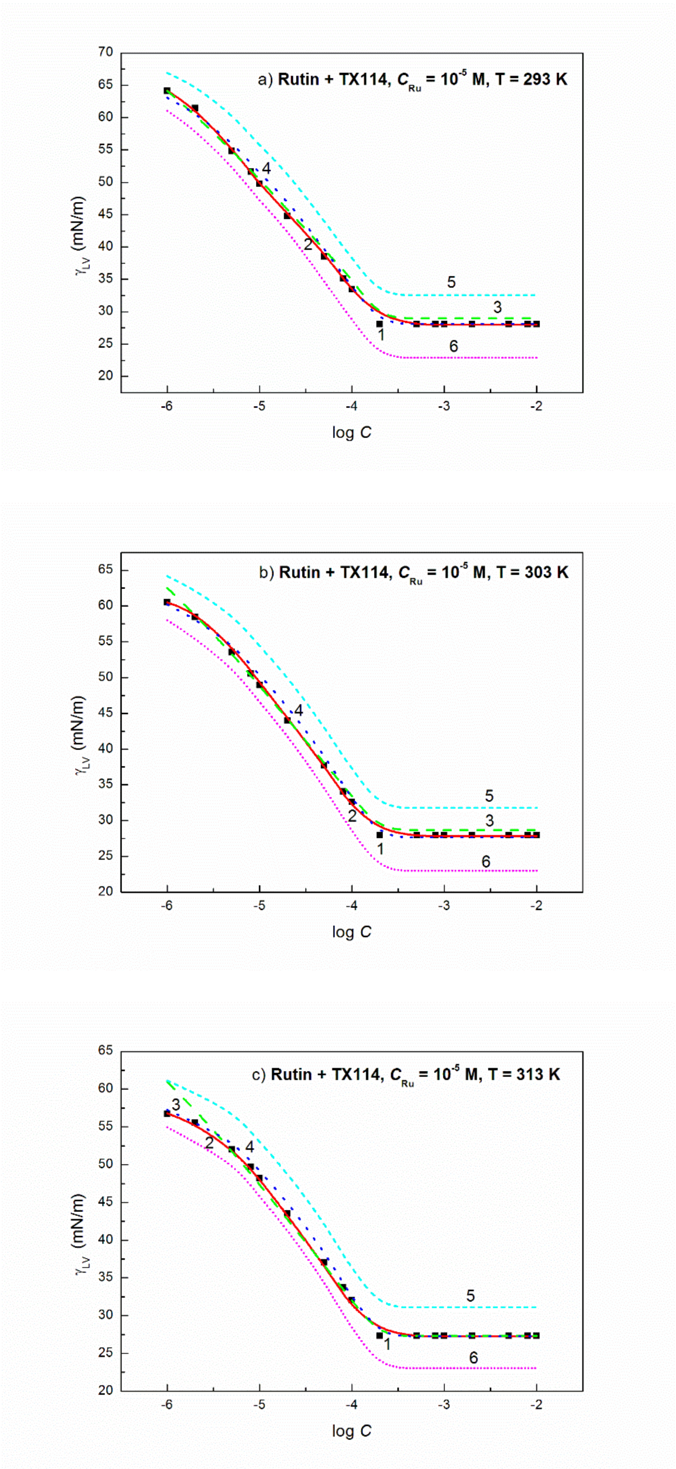

Figure S6. A plot of the surface tension (γ_{LV}) of aqueous solutions of Ru + TX114 at the constant Ru concentration equal to 1 × 10^{-5} M vs. the logarithm of TX114 concentration (C) at T = 293 K (a), 303 K (b) and 313 K (c). Points 1 correspond to the measured values. Curves 2, 3, 4, 5 and 6 correspond to the values calculated from Eqs. (4), (2), (6), (7) and independent adsorption, respectively.

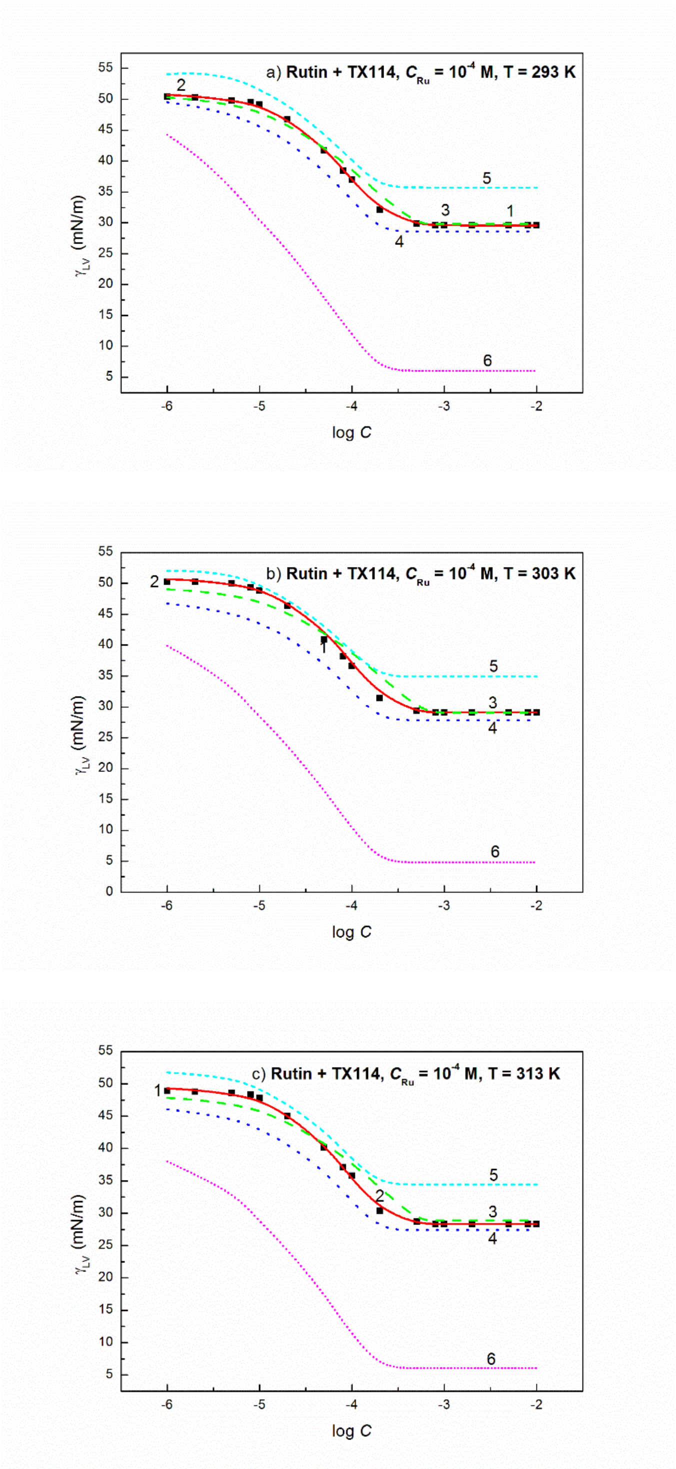

Figure S7. A plot of the surface tension (γ_{LV}) of aqueous solutions of Ru + TX114 at the constant Ru concentration equal to 1 × 10^{-4} M vs. the logarithm of TX114 concentration (C) at T = 293 K (a), 303 K (b) and 313 K (c). Points 1 correspond to the measured values. Curves 2, 3, 4, 5 and 6 correspond to the values calculated from Eqs. (4), (2), (6), (7) and independent adsorption, respectively.

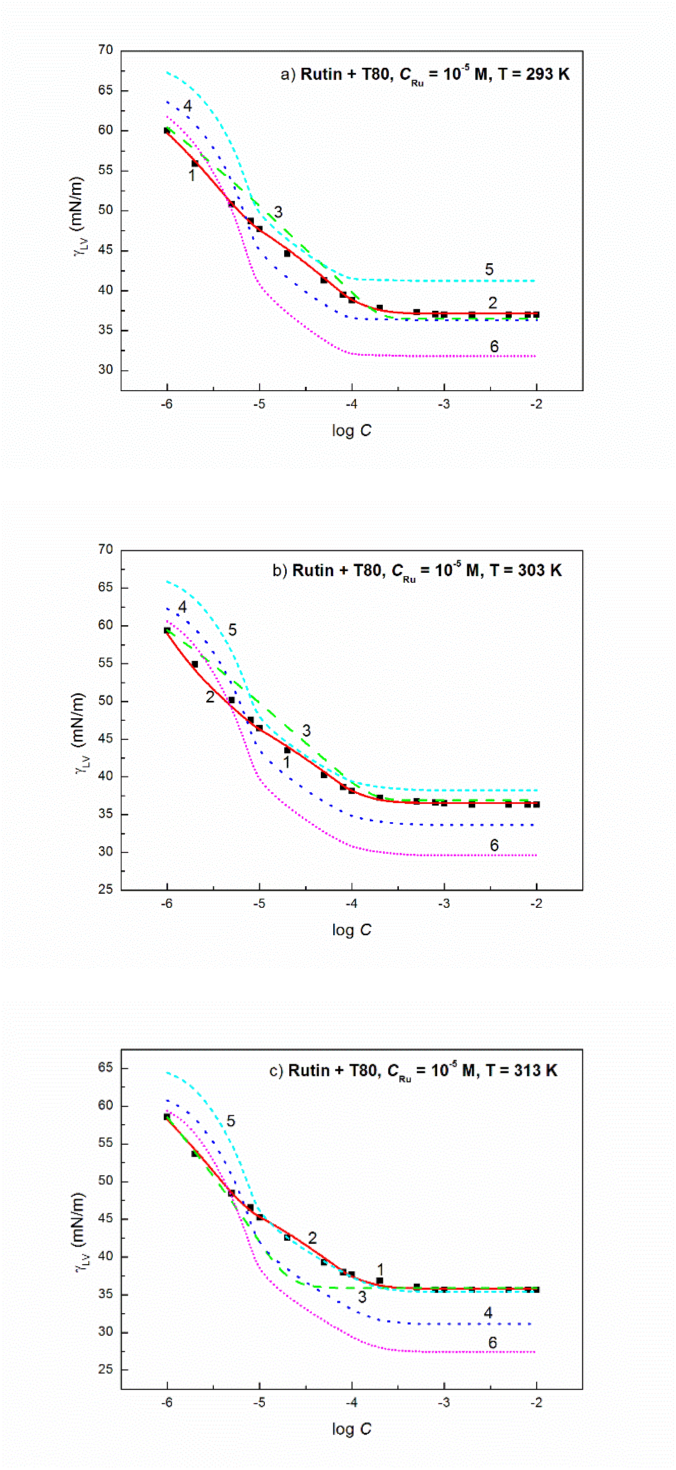

Figure S8. A plot of the surface tension (γ_{LV}) of aqueous solutions of Ru + T80 at the constant Ru concentration equal to 1 × 10^{-5} M vs. the logarithm of T80 concentration (C) at T = 293 K (a), 303 K (b) and 313 K (c). Points 1 correspond to the measured values. Curves 2, 3, 4, 5 and 6 correspond to the values calculated from Eqs. (4), (2), (6), (7) and independent adsorption, respectively.

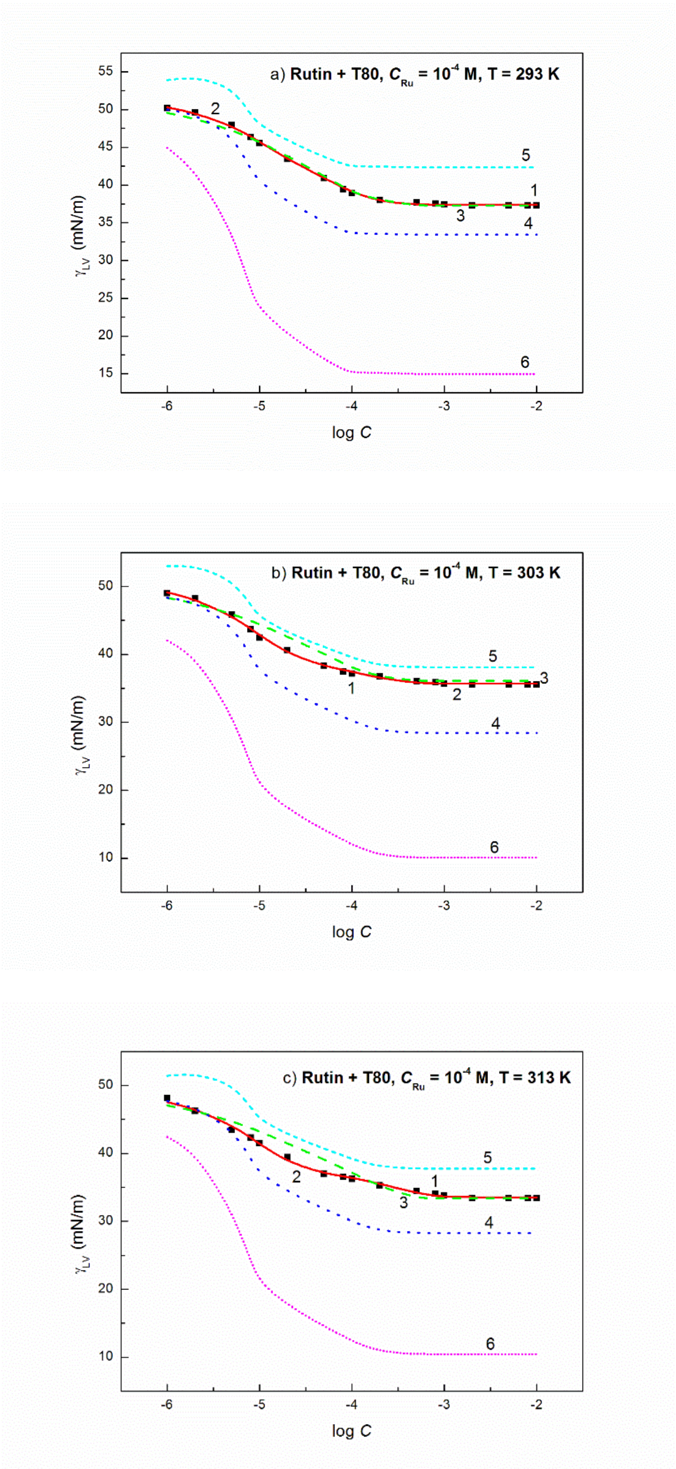

Figure S9. A plot of the surface tension (γ_{LV}) of aqueous solutions of Ru + T80 at the constant Ru concentration equal to 1 × 10^{-4} M vs. the logarithm of T80 concentration (C) at T = 293 K (a), 303 K (b) and 313 K (c). Points 1 correspond to the measured values. Curves 2, 3, 4, 5 and 6 correspond to the values calculated from Eqs. (4), (2), (6), (7) and independent adsorption, respectively.

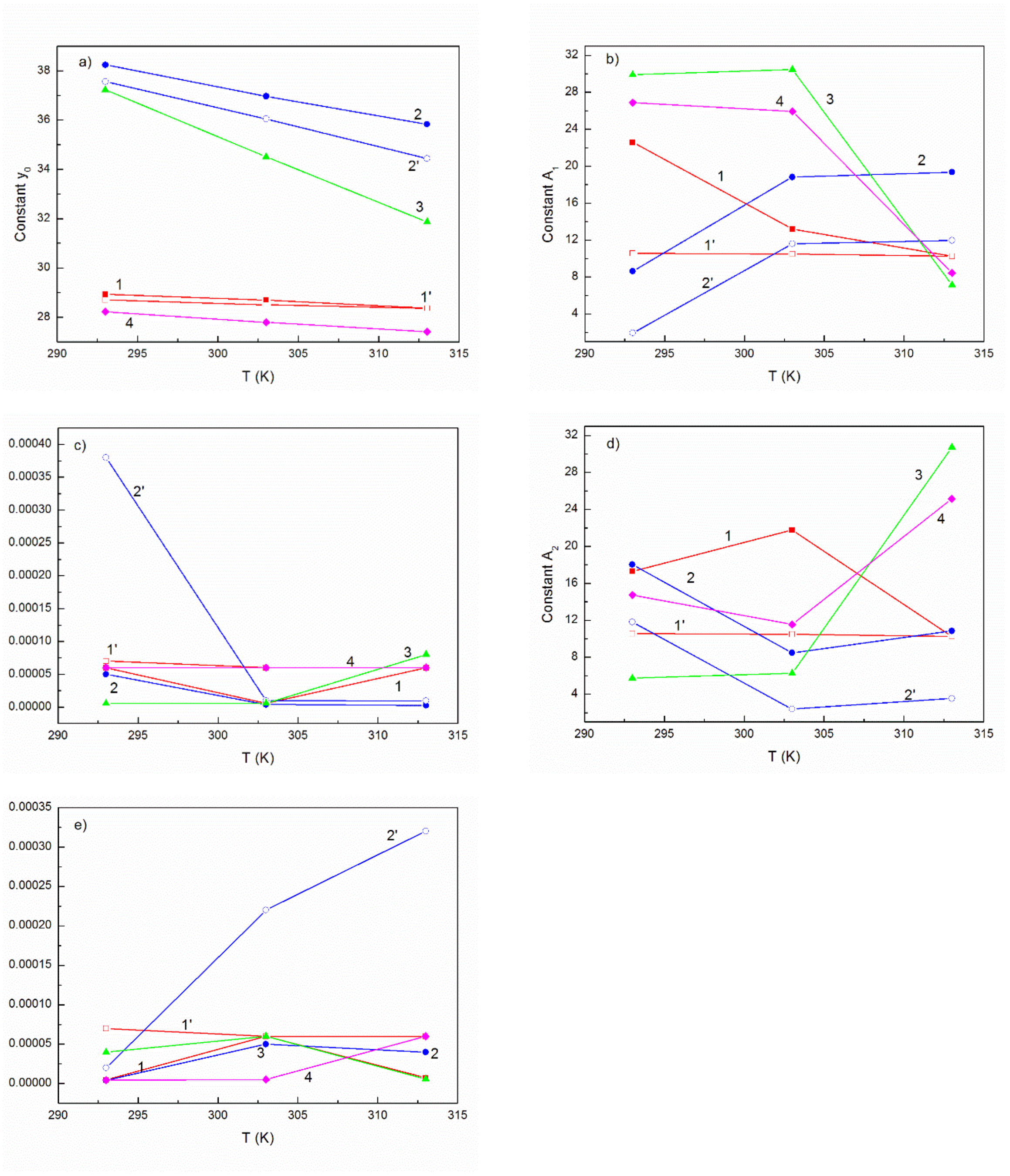

Figure S10. A plot of the constant y_0 (a), A_1 (b), t_1 (c), A_2 (d) and t_2 (e) in Eq. (4) for the Q + TX114 (curves 1 and 1') and Q + T80 (curves 2 and 2'), T80 (curve 3) and TX114 (curve 4) vs. the temperature (T). Curves 1 and 2 correspond to the constant flavonoid concentration equal to 1 × 10^{-5} M, curves 1' and 2' to the concentration equal to 1 × 10^{-4} M.

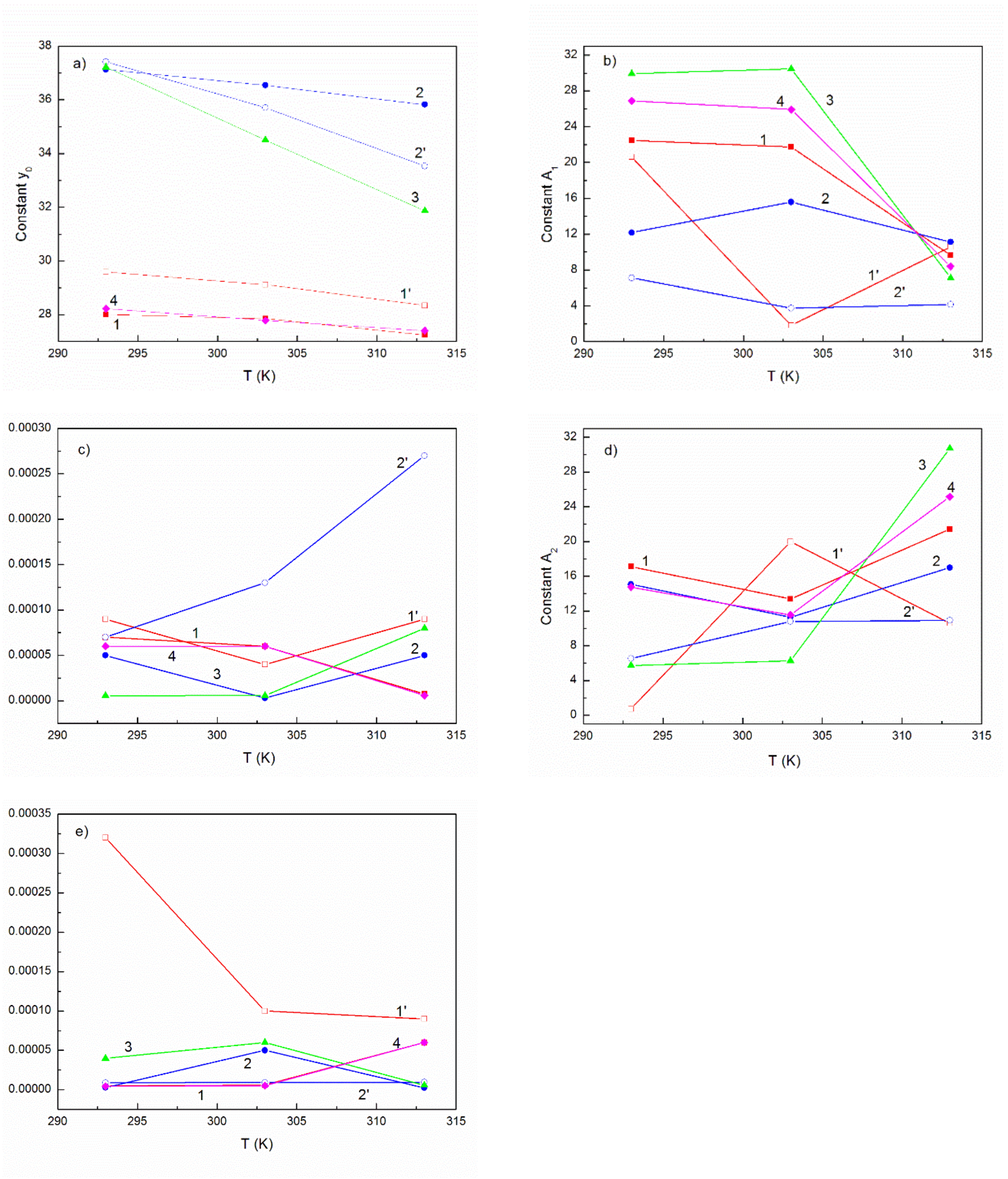

Figure S11. A plot of the constant y_0 (a), A_1 (b), t_1 (c), A_2 (d) and t_2 (e) in Eq. (4) for the Ru + TX114 (curves 1 and 1’) and Ru +T 80 (curves 2 and 2’), T80 (curve 3) and TX114 (curve 4) vs. the temperature (T). Curves 1 and 2 correspond to the constant flavonoid concentration equal to 1 × 10^{-5} M, curves 1’ and 2’ to the concentration equal to 1 × 10^{-4} M.

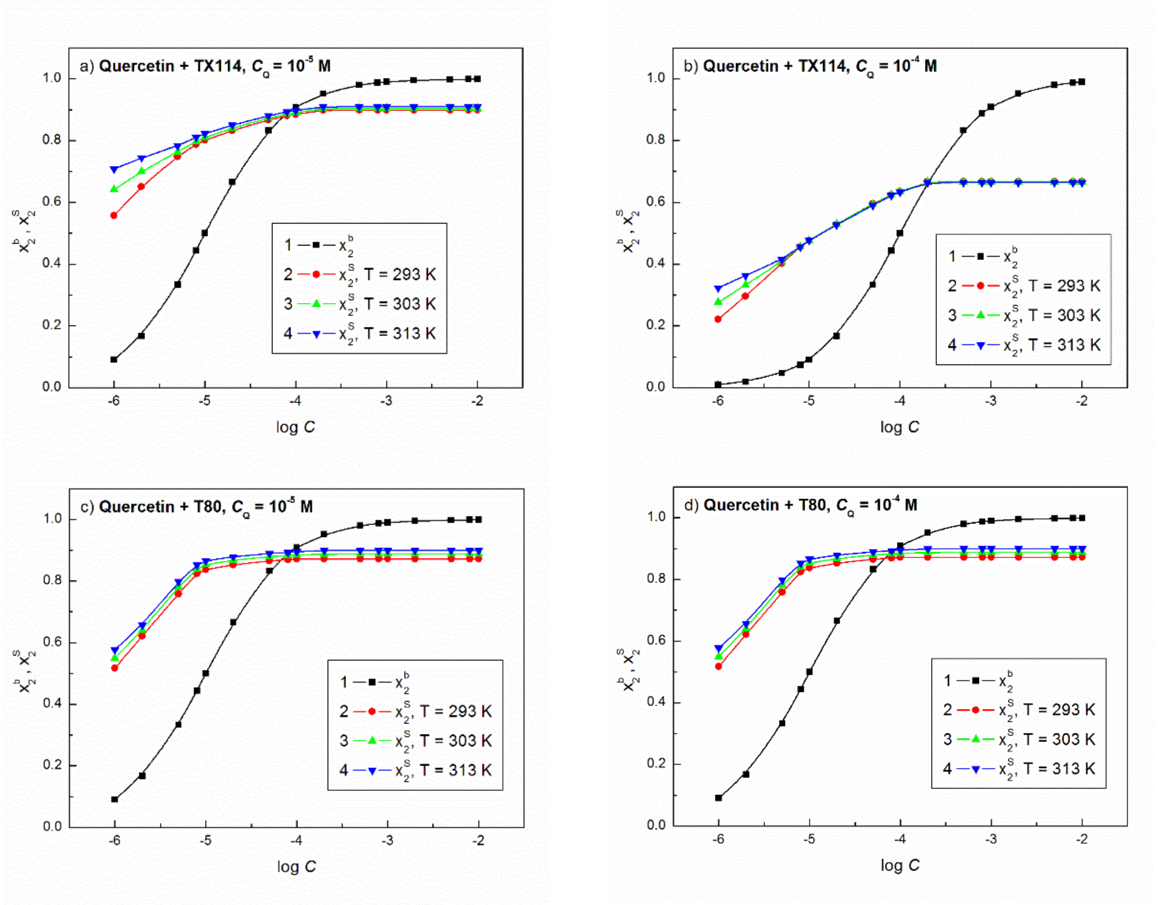

Figure S12. A plot of the values of x_2^b (curve 1) and x_2^s at temperature equal to 293 K (curve 2), 303 K (curve 3) and 313 K (curve 4) for aqueous solutions of Q + TX114 at the constant Q concentration equal to 1 × 10^{-5} M (a) and 1 × 10^{-4} M (b) as well as Q + T80 at the constant Q concentration equal to 1 × 10^{-5} M (c) and 1 × 10^{-4} M (d) vs. the logarithm of surfactant concentration (C).

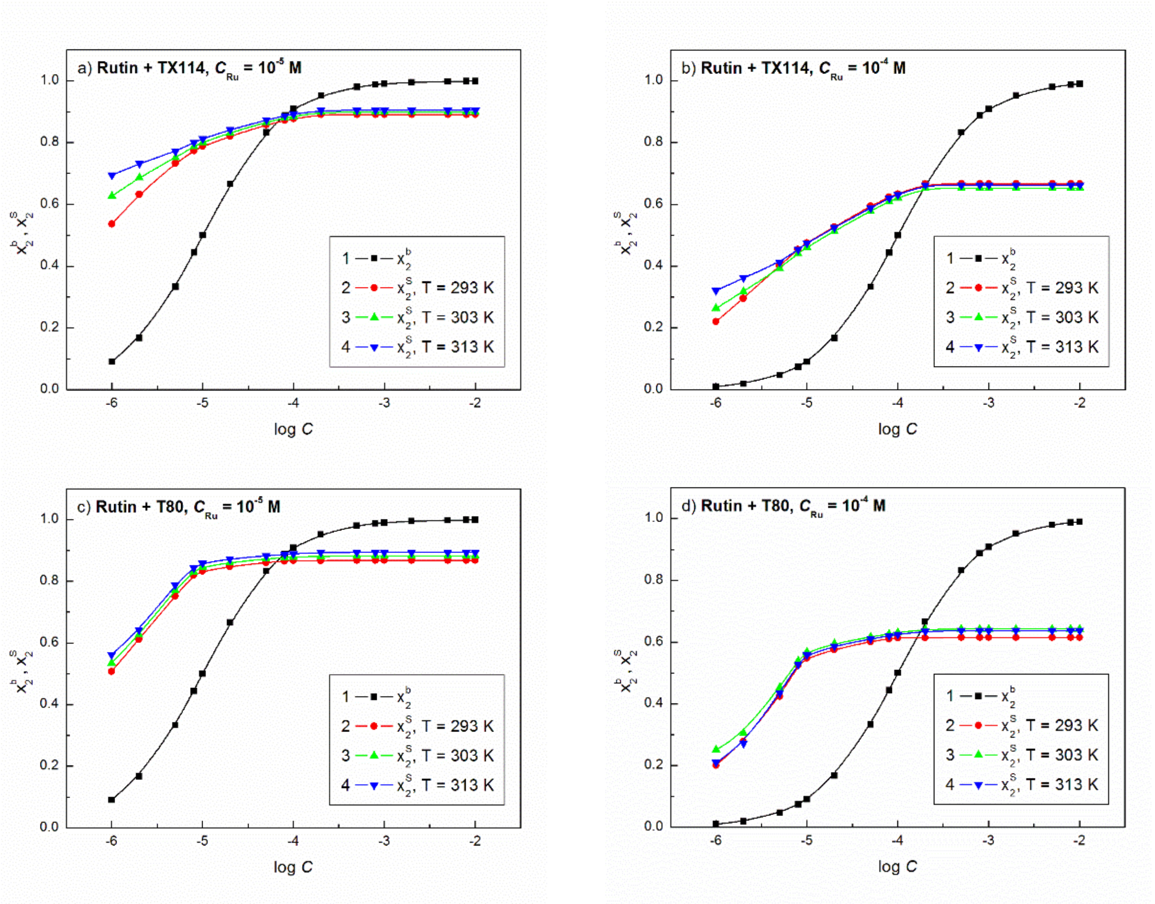

Figure S13. A plot of the values of x_2^b (curve 1) and x_2^s at temperature equal to 293 K (curve 2), 303 K (curve 3) and 313 K (curve 4) for aqueous solutions of Ru + TX114 at the constant Ru concentration equal to 1 × 10^{-5} M (a) and 1 × 10^{-4} M (b) as well as Ru + T80 at the constant Ru concentration equal to 1 × 10^{-5} M (c) and 1 × 10^{-4} M (d) vs. the logarithm of surfactant concentration (C).

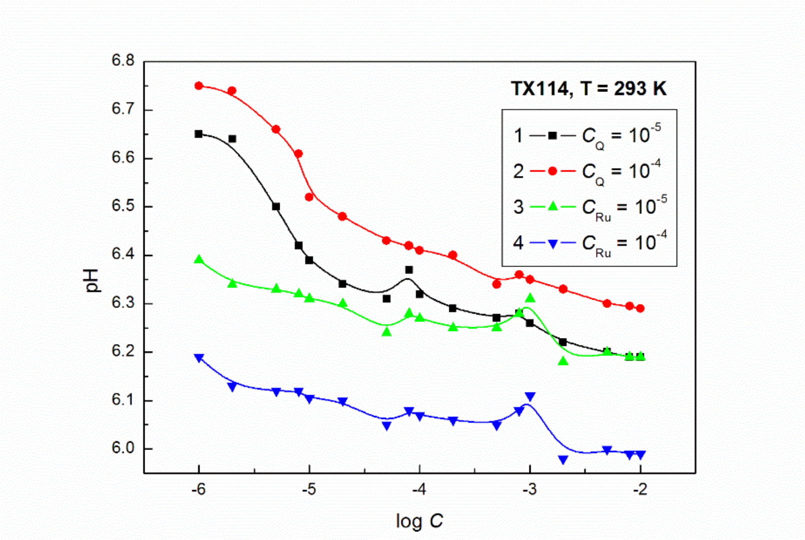

Figure S14. A plot of the values of pH of the aqueous solutions of TX114 with quercetin (curves 1 and 2) and rutin (curves 3 and 4) at temperature equal to 293 K vs. the logarithm of surfactant concentration (C).

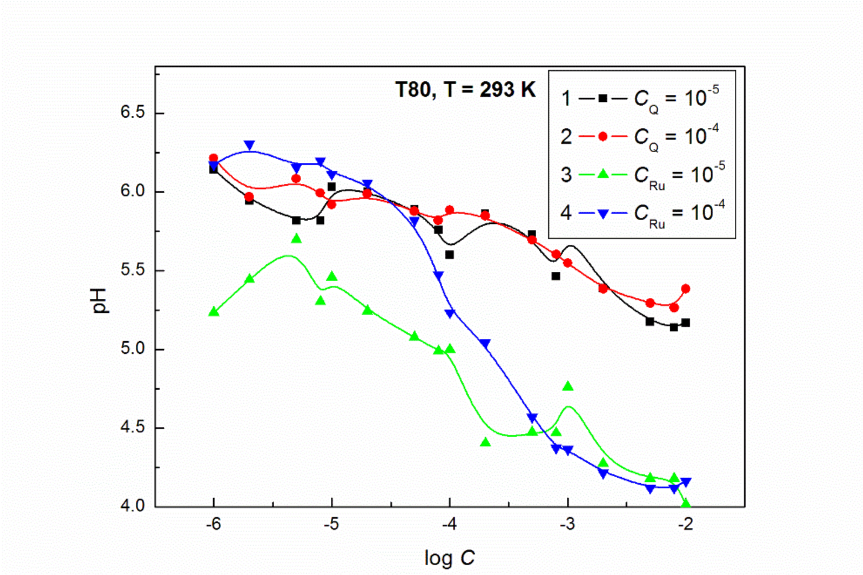

Figure S15. A plot of the values of pH of the aqueous solutions of T80 with quercetin (curves 1 and 2) and rutin (curves 3 and 4) at temperature equal to 293 K vs. the logarithm of surfactant concentration (C).

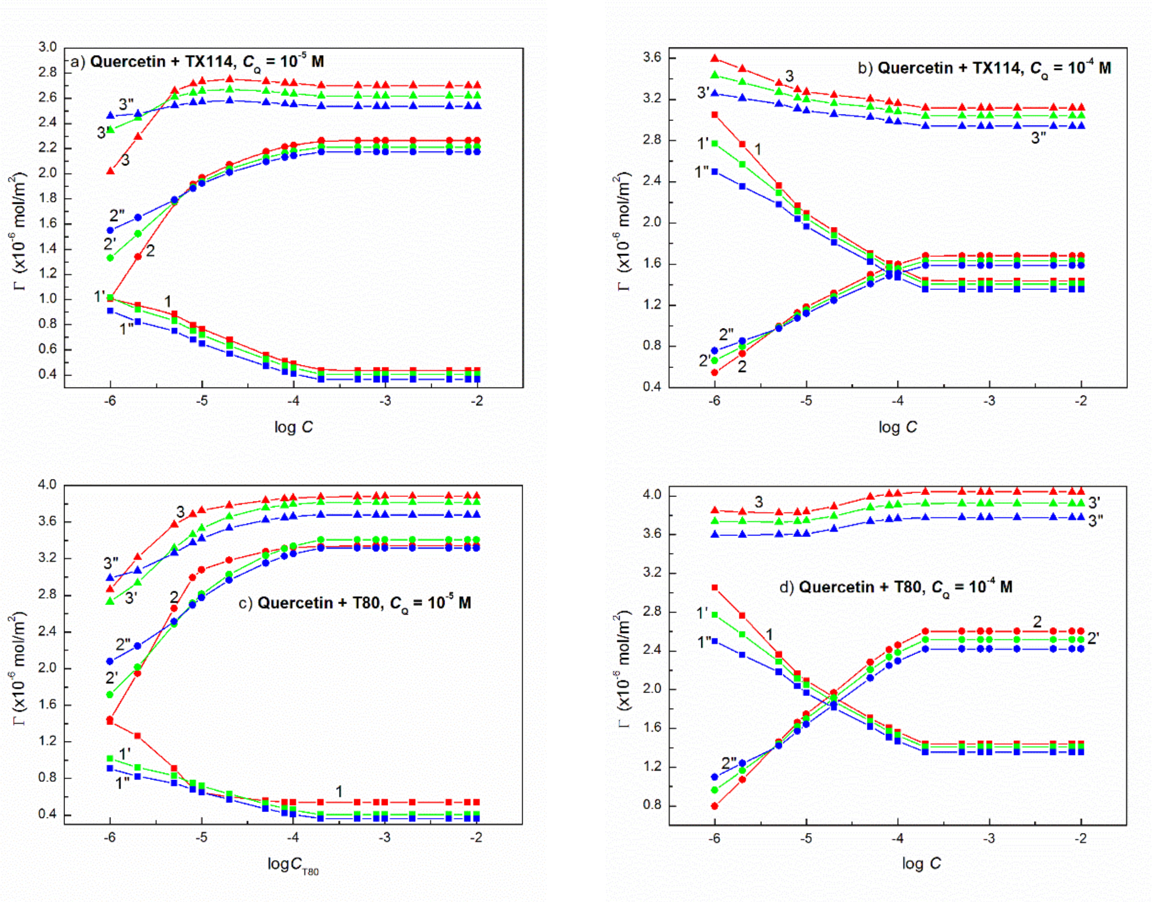

Figure S16. A plot of the surface concentration (Γ) of Q (curves 1' and 1"), TX114/ T80 (curves 2, 2' and 2") and the sum values for Q and TX114/T80 (curves 3, 3' and 3") calculated from Eq. (9) vs. the logarithm of TX114/T80 concentration (*C*) at the constant Q concentration equal to 1 × 10^{-5} M ((a) and c)) and 1 × 10^{-4} M ((b) and (d)). Curves 1, 2 and 3 correspond to temperature equal to 293 K. curves 1', 2' and 3' to 303 K and curves 1", 2" and 3" to 313 K, respectively.

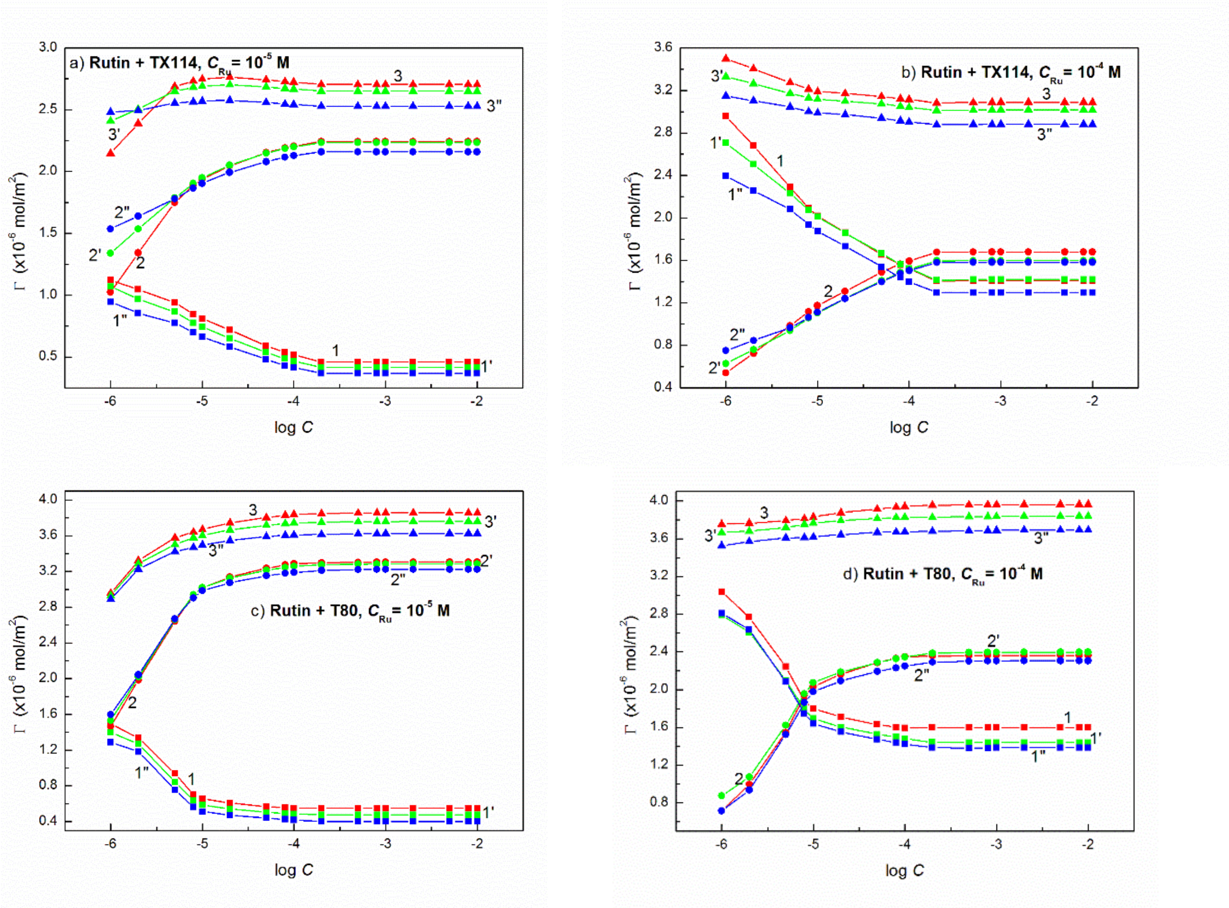

Figure S17. A plot of the surface concentration (Γ) of Ru (curves 1' and 1''), TX114/ T80 (curves 2, 2' and 2'') and the sum values for Ru and TX114/T80 (curves 3, 3' and 3'') calculated from Eq. (9) vs. the logarithm of TX114/T80 concentration (*C*) at the constant Ru concentration equal to 1 × 10^{-5} M ((a) and c)) and 1 × 10^{-4} M ((b) and (d)). Curves 1, 2 and 3 correspond to temperature equal to 293 K. curves 1', 2' and 3' to 303 K and curves 1'', 2'' and 3'' to 313 K, respectively.
